# Nectin cell adhesion molecule 4 regulates angiogenesis through Src signaling and serves as a novel therapeutic target in angiosarcoma

**DOI:** 10.1038/s41598-022-07727-x

**Published:** 2022-03-07

**Authors:** Yuka Tanaka, Maho Murata, Keiko Tanegashima, Yoshinao Oda, Takamichi Ito

**Affiliations:** 1 Department of Dermatology, Graduate School of Medical Sciences, Kyushu University, 3-1-1 Maidashi, Higashi-ku, Fukuoka City, Fukuoka 812-8582 Japan; 2 Department of Anatomic Pathology, Graduate School of Medical Sciences, Kyushu University, Fukuoka, Japan

**Keywords:** Skin cancer, Cell biology

## Abstract

Angiosarcoma is a rare, life-threatening soft tissue sarcoma with malignant endothelial cells that is mainly found in the skin. Multidisciplinary approaches are used to treat patients with unresectable metastasized lesions; considering the cellular origin of angiosarcoma, anti-angiogenic therapy has also been used recently. However, these treatments have limited efficacy, and the survival rate remains low. Thus, more effective treatments need to be developed. Nectin cell adhesion molecule 4 (NECTIN4) is highly expressed in malignant tumors and promotes tumor progression. Thus, NECTIN4 is expected to be a novel therapeutic target for cancer. However, the significance of NECTIN4 in angiosarcoma remains unknown. Using immunohistochemistry, we investigated NECTIN4 expression in 74 tissue samples from angiosarcoma patients, finding variable NECTIN4 expression. In addition, we investigated NECTIN4 expression and function in human angiosarcoma cell lines. NECTIN4 expression was higher in angiosarcoma cells than normal endothelial cells, and angiosarcoma cells were sensitive to monomethyl auristatin E, the cytotoxic part of a NECTIN4-targetting antibody–drug conjugate. NECTIN4 knockdown inhibited the proliferation and angiogenesis of angiosarcoma cells, and Src kinase signaling was shown to be involved in NECTIN4 function, at least in part. NECTIN4-targeted therapy has the potential to be a novel treatment strategy for angiosarcoma.

## Introduction

Angiosarcoma is a rare soft tissue sarcoma with malignant endothelial cells derived from blood or lymphatic vessels^1,2^. Angiosarcoma accounts for 1–2% of soft tissue sarcoma, and is commonly found as an asymptomatic and sporadic lesion of the scalp or face^2,3^. Angiosarcoma is most commonly found in elderly men, with previous radiation therapy and chronic lymphedema being well-known risk factors^4^. Angiosarcoma has an aggressive nature, and the lesions become nodular with fungation, ulceration, and hemorrhage when developed. Tumor cells tend to spread by either lymphatic or hematogenous routes, with a 5-year survival rate of approximately 31% and a median survival of 7 months for patients with metastatic disease^3^. At present, surgical resection is the primary treatment of choice for resectable angiosarcoma, with adjuvant radiotherapy and chemotherapy (paclitaxel, taxane, anthracycline) with/without radiotherapy is used for the treatment of unresectable disease^5–8^. Considering the origin of angiosarcoma cells, attention is now focusing on anti-angiogenic therapy, such as the anti-vascular endothelial growth factor A (VEGFA) monoclonal antibody bevacizumab and the broad-spectrum tyrosine-kinase inhibitors sorafenib and pazopanib, which target vascular endothelial growth factor receptors (VEGFRs). Pazopanib, a multitarget tyrosine kinase inhibitor approved for soft tissue sarcoma, was tested in angiosarcoma, with some patients responding to treatment^9,10^. However, the efficacy of these treatments is limited and the response rate remains poor. In several reports, the overall response rate of angiosarcoma to anti-angiogenic therapy was reported to be only 0–14%, with a median overall survival of approximately 14 months^1^. Thus, there is a need to develop more effective treatments for angiosarcoma.

Nectin cell adhesion molecule 4 (NECTIN4; also known as poliovirus receptor-related protein 4 [PVRL4]) is one of the cell adhesion molecules found in adherens junctions^11^. NECTIN4 is a member of the nectin family, which regulates various cell functions, such as cell polarity, proliferation, differentiation, migration, and invasion^12,13^. NECTIN4 is also known to function in tumors. It is highly expressed in several types of cancers, such as urothelial, breast, and lung cancers, and promotes the proliferation of cancer cells by activating the phosphatidylinositol-3 kinase (PI3K)/Akt pathway^14–16^. NECTIN4 also induces epithelial-to-mesenchymal transition (EMT), which contributes to the metastasis of tumor cells^15^. Thus, NECTIN4 is now attracting attention as a potential target for cancer treatment. Indeed, using a novel type of targeted drug, namely antibody–drug conjugates (ADCs), NECTIN4-targeted therapy has been tested in several clinical trials. The efficacy of treatment with enfortumab vedotin, a NECTIN4-targeted ADC, has been assessed in urothelial cancer^17,18^ and in locally advanced or metastatic malignant solid tumors (EV-202; NCT04225117)^19^. In normal skin tissue, NECTIN4 is found in the epidermis and in skin appendages such as sweat glands and hair follicles^20–22^. NECTIN4 is also expressed in diseased skin, such as extramammary Paget’s disease (EMPD), squamous cell carcinoma, and melanoma^23–25^. We recently reported that high NECTIN4 expression is associated with poor prognosis in EMPD and melanoma^23,24^. However, the expression and function of NECTIN4 in angiosarcoma remains unknown.

The aims of the present study were to reveal the expression and function of NECTIN4 in angiosarcoma and to assess the utility of NECTIN4 as a target of angiosarcoma treatment. To this end, we assessed NECTIN4 expression in angiosarcoma lesions of patients. NECTIN4 is also expressed in the HAMON and the ISO-HAS-B angiosarcoma cell lines, with NECTIN4 expression higher in angiosarcoma cells than normal endothelial cells. Further in vitro analyses revealed the involvement of NECTIN4 in the regulation of tumor cell proliferation, apoptosis, and angiogenesis. Our findings demonstrate the expression and function of NECTIN4 in angiosarcoma and suggest the utility of NECTIN4 as a novel therapeutic target for the treatment of angiosarcoma.

## Results

### NECTIN4 expression in human angiosarcoma lesions

**Figure 1:**
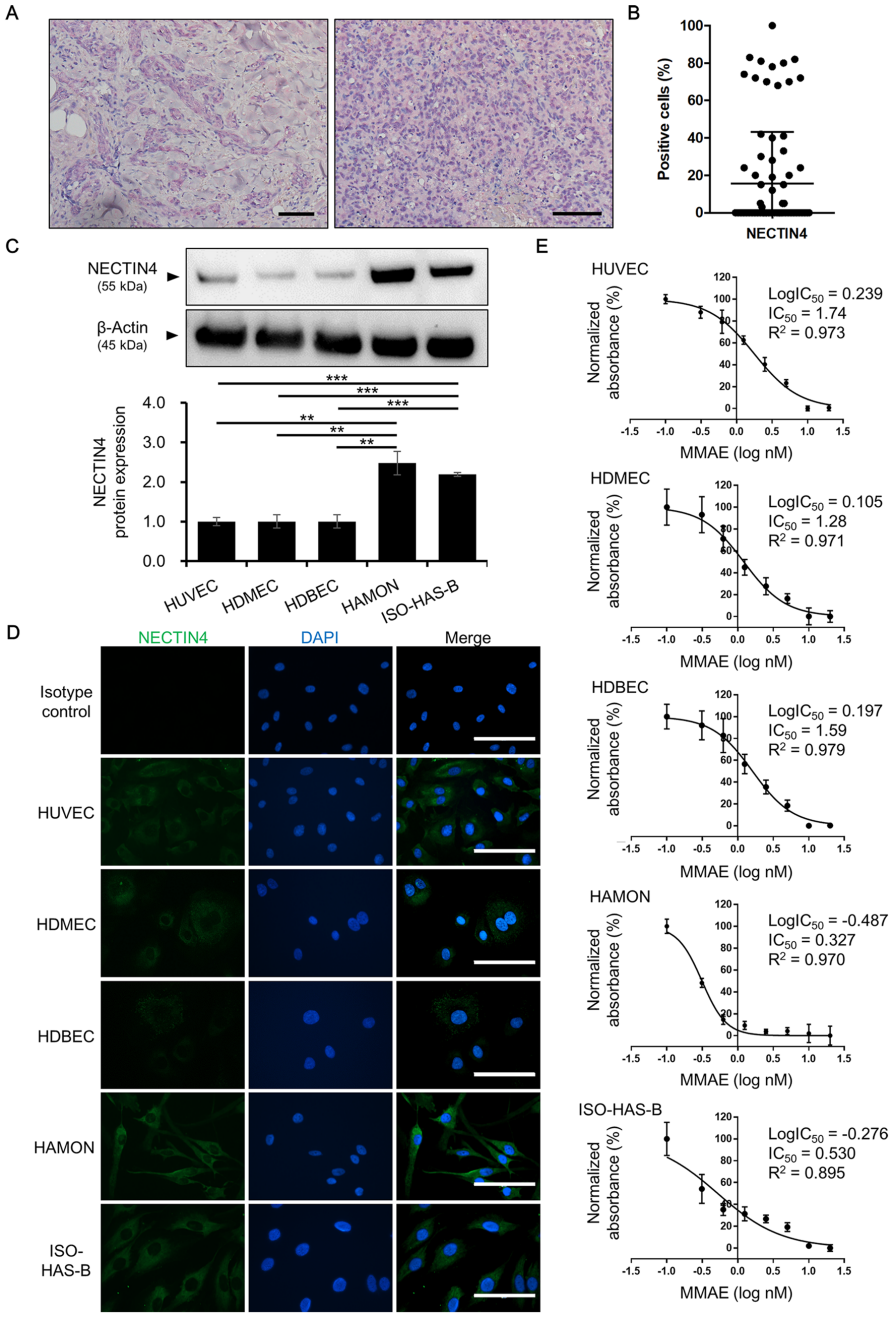
NECTIN4 expression in angiosarcoma. (**A**) Representative images of NECTIN4 staining (red) of samples taken from angiosarcoma patients. Scale bars = 100 μm. (**B**) Percentage of NECTIN4-positive tumor cells in samples from 74 angiosarcoma patients. Data are the mean ± SD. (**C**) Representative blots showing NECTIN4 and β-actin expression in HUVEC, HDMEC, HDBEC, HAMON, and ISO-HAS-B cells and mean (± SD) NECTIN4 protein expression in three independent experiments. The NECTIN4 signal was analysed using ImageJ software and was normalized against that of β-actin. The images shown derive from the same experiment and the blots were processed in parallel. Original blot images are presented in Supplementary Fig. S2. (**D**) Representative images of immunocytochemical detection of NECTIN4 in HUVEC, HDMEC, HDBEC, HAMON, and ISO-HAS-B cells. Nuclei were stained with DAPI. Scale bars = 100 μm. (**E**) Dose–response curve of monomethyl auristatin E (MMAE) in HUVEC, HDMEC, HDBEC, HAMON, and ISO-HAS-B cells treated for 48 h with DMSO (0.1%) or different concentrations (0.1–20.0 nM) of MMAE. Mean of LogIC50, IC50, and R2 was indicated in upper right. Experiments were repeated three times with three wells for each concentration, and shown as mean (± SD).

Immunohistochemical staining of NECTIN4 was used to investigate NECTIN4 expression in human angiosarcoma lesions. The mean age of patients was 70.42 years (range 40–92 years), and 58.11% were male. The primary tumor site was the skin (71.62%), followed by the liver (6.76%), bone (5.41%), soft tissue (5.41%), spleen (4.05%), adrenal glands (2.70%), gastrointestinal tract (2.70%), and heart (1.35%). Immunohistochemical staining showed variable expression of NECTIN4 in tumor cells (Fig. 1A). The proportion of NECTIN4-positive tumor cells varied among patients, ranging from 0 to 100% (Fig. 1B). We further analyzed the relationship between clinical factors and NECTIN4 expression (Supplementary Table S1). There was no significant correlation between NECTIN4 expression and age or sex. Of note, positive NECTIN4 cases were more common in tumors with high-grade malignant potential (i.e. non-skin angiosarcoma or epithelioid histopathological subtype), although the correlation was statistically not significant.

### NECTIN4 expression in human angiosarcoma cell lines

Because NECTIN4 was expressed in angiosarcoma tissue, we next investigated the functions of NECTIN4 in angiosarcoma using the HAMON and the ISO-HAS-B angiosarcoma cell lines. NECTIN4 expression in tumor cells was compared with that in normal human endothelial cells (i.e. human umbilical vein endothelial cells [HUVEC], human dermal microvascular endothelial cells [HDMEC], and human dermal blood endothelial cells [HDBEC]). NECTIN4 mRNA and protein was expressed in both normal endothelial cells and angiosarcoma cells, but was significantly higher in angiosarcoma cells (Fig. 1C; Supplementary Fig. S1, S2). Immunocytochemistry revealed stronger NECTIN4 staining intensity in angiosarcoma cells relative to normal endothelial cells (Fig. 1D). Expression of cyclin D1 (*CCND1*), a cell proliferation marker, *VEGFA* and *VEGF*_*165*_ (a splice variant of *VEGFA*), potent inducers of angiogenesis, was significantly higher in angiosarcoma cells than in normal endothelial cells and HAMON cells showed higher expression than ISO-HAS-B (Supplementary Fig. S3). Expression of *BCL-2*, an anti-apoptotic factor, was significantly high in HAMON cells, but not in ISO-HAS-B.

### Sensitivity of angiosarcoma cells to monomethyl auristatin E

We also evaluated the effects of the mitosis inhibitor monomethyl auristatin E (MMAE), which is the cytotoxic part of a NECTIN4-targeted ADC, on the viability of normal endothelial cells (HUVEC, HDMEC, and HDBEC) and angiosarcoma cells (HAMON and ISO-HAS-B). The test concentrations of MMAE used in this study were determined on the basis of the MMAE concentration in peripheral blood reported previously^17^. After 48 h exposure to MMAE, the viability of MMAE-treated angiosarcoma cell lines was significantly lower than that of dimethylsulfoxide (DMSO)-treated control cells, even at a concentration of 0.313 nM MMAE, the concentration that is much lower than the concentration in peripheral blood (Fig. 1E). IC50 of MMAE in angiosarcoma cell lines were significantly lower than that in normal endothelial cells (Fig. 1E) (IC50 = 1.74 ± 0.150, 1.28 ± 0.134, 1.59 ± 0.289, 0.32 7 ± 0.0394, and 0.530 ± 0.0189 in HUVEC, HEMEC, HDBEC, HAMON, and ISO-HAS-B, respectively), indicating that these angiosarcoma cells are more sensitive to MMAE than normal endothelial cells.

### Effects of NECTIN4 knockdown on the proliferation of angiosarcoma cells

**Figure 2:**
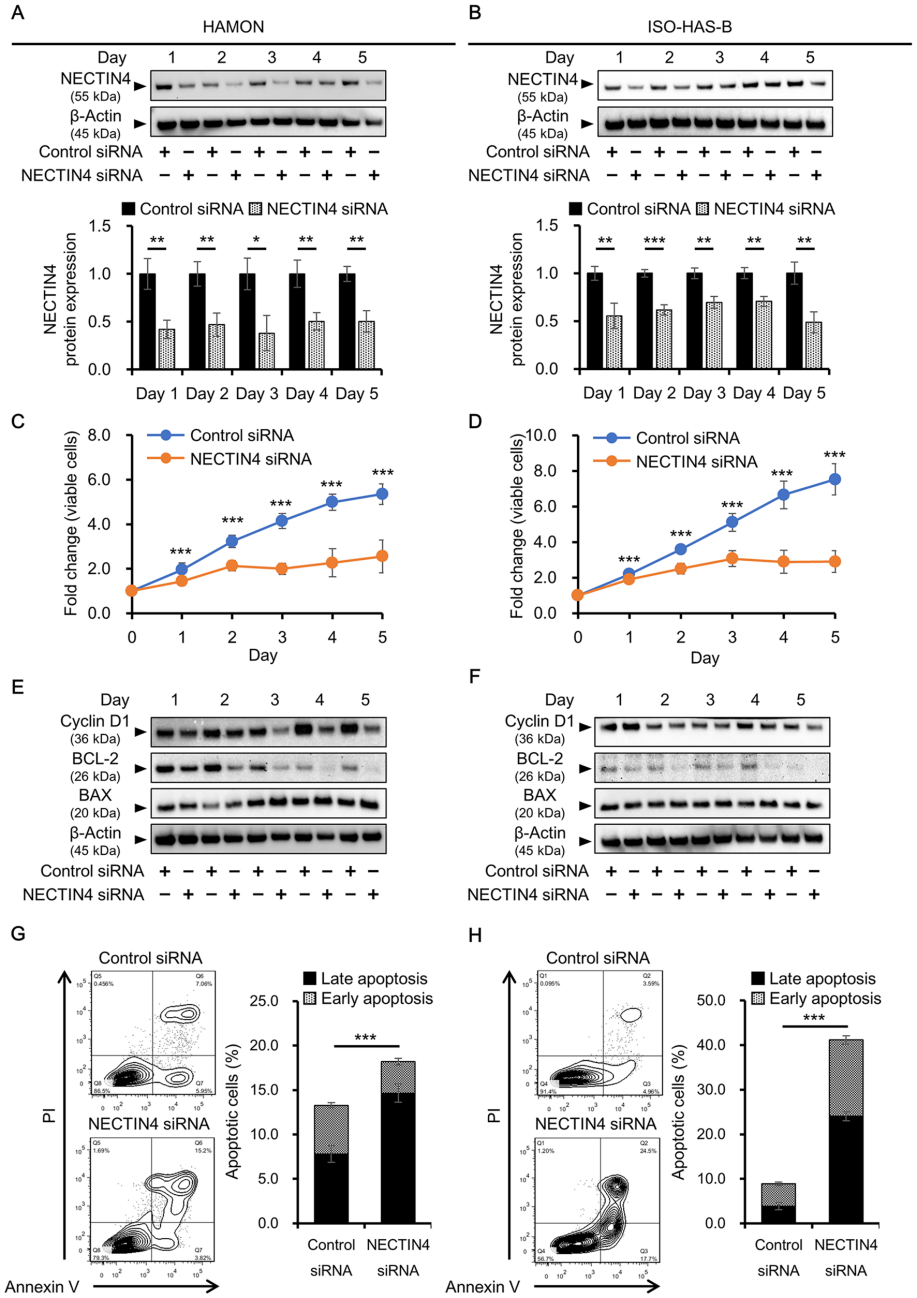
Knockdown of NECTIN4 downregulates angiosarcoma cell proliferation and induces apoptosis. HAMON and ISO-HAS-B cells were transfected with control or NECTIN4 siRNA and assessed for the number of viable cells, expression of related molecules, and apoptosis. (**A,B**) Mean (± SD) NECTIN4 knockdown efficiency at protein level in (**A**) HAMON and (**B**) ISO-HAS-B cells determined in three independent experiments. Representative blot images of NECTIN4 protein expression are also shown. The NECTIN4 signal was analysed using ImageJ software and was normalized against that of β-actin. The images shown derive from the same experiment and the blots were processed in parallel. Full-length blots are presented in Supplementary Fig. S4. (**C,D**) Mean (± SD) number of viable cells in siRNA-transfected (**C**) HAMON and (**D**) ISO-HAS-B cells, as detected by the CCK-8 assay. Data show fold changes relative to Day 0. Experiments were repeated three times, with five wells used for each concentration. (**E,F**) Representative blot images of cyclin D1, BCL-2, BAX, and β-actin in siRNA-transfected (**E**) HAMON and (**F**) ISO-HAS-B cells. The images shown derive from the same experiment and the blots were processed in parallel. Full-length blots are presented in Supplementary Fig. S6 and S7. Membranes were cut according to the molecular weight to separately hybridize with different antibodies. (**G,H**) Representative counter plot images of Annexin V-PI staining and the percentages of apoptotic cells in (**G**) HAMON and (**H**) ISO-HAS-B cells. Percentages of apoptotic cells among total cells were calculated from the results of three independent experiments. *P < 0.05, **P < 0.01, and *** *P* < 0.001 compared with control siRNA-transfected cells.

Next, we investigated the functions of NECTIN4 in angiosarcoma cells by knocking down NECTIN4 using short interference (si) RNA. Knockdown efficiency at the NECTIN4 mRNA and protein levels was confirmed by quantitative reverse transcription–polymerase chain reaction (qRT-PCR) and western blotting, respectively (Fig. 2A,B; Supplementary Figs S4, S5). When NECTIN4 expression was suppressed by siRNA, the number of viable cells was significantly reduced compared with that in control siRNA-transfected cells, with a 52.3 ± 13.7% reduction in viability by Day 5 in HAMON (Fig. 2C) and 61.4 ± 8.08% reduction in ISO-HAS-B (Fig. 2D). We further analyzed the expression of cyclin D1 (a cell proliferation marker), BCL-2 (an anti-apoptotic factor), and BAX (an apoptosis inducer), all of which affect cell viability (Fig. 2E,F; Supplementary Figs S5–7). Cyclin D1 was significantly decreased by NECTIN4 siRNA transfection in HAMON cells (Fig. 2E; Supplementary Figs S5, S6) and in ISO-HAS-B cells (Fig. 2F; Supplementary Figs S5, S7). Cell cycle was also analyzed by propidium iodide (PI) staining and flow cytometry (Supplementary Fig. S8A). In HAMON cells, knockdown of NECTIN4 significantly increased G0/G1 phase and decreased S phase (Supplementary Fig. S8B, left panels). Same tendency was observed in ISO-HAS-B, but the differences were not statistically significant (Supplementary Fig. S8B, right panels).

When apoptosis-related factors were analyzed, we found that BCL-2 expression was significantly decreased in NECTIN4 siRNA-transfected cells compared with control siRNA-transfected HAMON (Fig. 2E; Supplementary Figs S5, S6) and in ISO-HAS-B (Fig. 2F; Supplementary Figs S5, S7). BAX expression was significantly induced in NECTIN4 siRNA-transfected HAMON and ISO-HAS-B cells (Fig. 2E,F; Supplementary Figs S5–7). Apoptotic status of NECTIN4 siRNA-transfected cells was further assessed by flow cytometry. Using PI and fluorescein (FITC)-conjugated Annexin V, apoptotic cells can be detected as early apoptotic (Annexin V-positive, PI-negative) cells and late apoptotic (Annexin V-positive, PI-positive) cells (Supplementary Fig. S8C). Annexin V-PI staining showed that apoptosis was significantly induced in NECTIN4-siRNA transfected HAMON and ISO-HAS-B (Fig. 2G,H). These results imply that NECTIN4 affects the number of viable angiosarcoma cells by modulating the expression of cyclin D1, BCL-2, and/or BAX.

### Effects of NECTIN4 knockdown on angiogenesis of angiosarcoma cells

**Figure 3:**
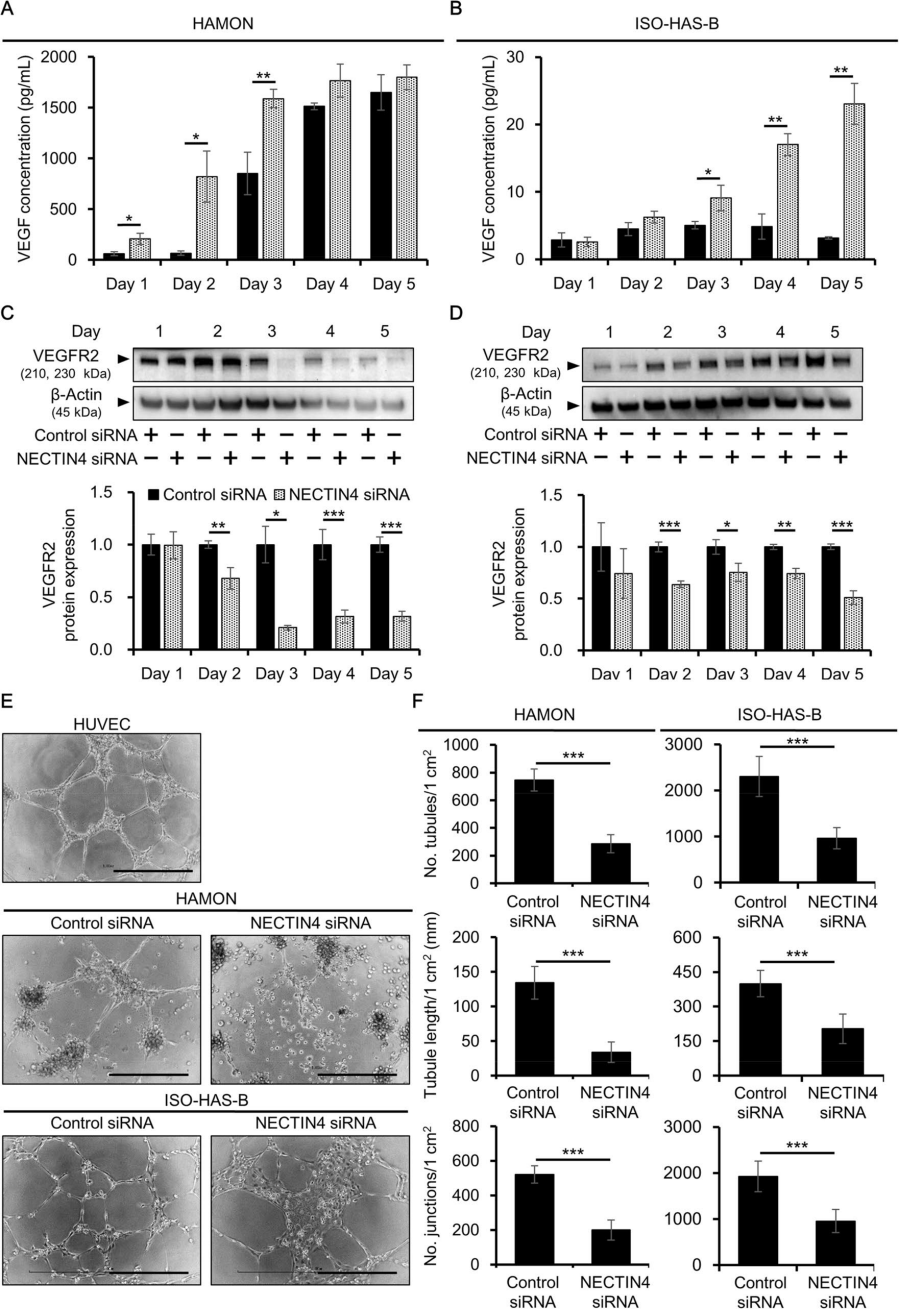
Knockdown of NECTIN4 prevents angiogenesis of angiosarcoma cells. HAMON and ISO-HAS-B cells were transfected with control or NECTIN4 siRNA and assessed for angiogenesis. (**A,B**) VEGF protein concentration in the culture supernatant of control or NECTIN4 siRNA-transfected (**A**) HAMON and (**B**) ISO-HAS-B cells. Data are the mean ± SD of three independent experiments. (**C,D**) Expression of VEGFR2 protein in control and NECTIN4 siRNA-transfected (**C**) HAMON and (**D**) ISO-HAS-B cells. Data are the mean ± SD of three independent experiments. Representative blot images are also shown. The VEGFR2 signal was analysed using ImageJ software and was normalized against that of β-actin. The images shown derive from the same experiment and the blots were processed in parallel. Membranes were cut according to the molecular weight to separately hybridize with different antibodies and original blot images are presented in Supplementary Fig. S9. (**E**) Representative images of the angiogenesis assay. HUVEC served as a positive control for tubule formation. Scale bars = 0.5 mm. (**F**) Number of tubules per 1 cm^2^, tubule length per 1 cm^2^, and the number of junctions per 1 cm^2^ for control and NECTIN4 siRNA-transfected HAMON (left) or ISO-HAS-B (right) cells. Data are the mean ± SD of three independent experiments. *P < 0.05, **P < 0.01, and ***P < 0.001 compared with control siRNA-transfected cells.

Angiogenesis is a process by which new blood vessels are formed from existing vessels, and it is known to be promoted in various cancers, including angiosarcoma^26,27^. NECTIN4 reportedly contributes to angiogenesis in some tumors, such as pancreatic cancer, but the function of NECTIN4 in angiogenesis in angiosarcoma has not yet been elucidated^28^. Thus, in the present study, we first evaluated the expression of VEGF, a growth factor that plays important roles in angiogenesis. We assessed VEGF protein concentrations in the culture supernatant of siRNA-transfected cells using enzyme-linked immunosorbent assay (ELISA). The production of VEGF was significantly and strongly induced in NECTIN4 siRNA-transfected HAMON cells compared with control siRNA-transfected cells 1–3 days after transfection, with 3.42 ± 0.92-, 12.4 ± 3.8-, and 1.87 ± 0.11-fold increases, respectively (Fig. 3A). In ISO-HAS-B, the production of VEGF was significantly induced in NECTIN4 siRNA-transfected cells compared with control siRNA-transfected cells 3–5 days after transfection, with 1.80 ± 0.37-, 3.50 ± 0.34-, and 7.27 ± 0.95-fold increases, respectively (Fig. 3B). We also assessed the expression of VEGFR2. In NECTIN4 siRNA-transfected cells, there was a significant downregulation in VEGFR2 protein expression in NECTIN4 siRNA-transfected cells 2–5 days after transfection in HAMON (Fig. 3C; Supplementary Fig. S9A) and in ISO-HAS-B (Fig. 3D; Supplementary Fig. S9B).

Given the upregulation in VEGF and downregulation in VEGFR2 in NECTIN4 siRNA-transfected cells, an in vitro tube formation assay was performed to evaluate angiogenesis in cells in which NECTIN4 was knocked down. In this assay, HUVEC were used as a positive control because they are able to form tubular structures on extracellular matrix (ECM) gels. Both HUVEC and control siRNA-transfected angiosarcoma cells formed tubular structure on ECM gels, but tube formation was obviously inhibited in NECTIN4-siRNA transfected HAMON and ISO-HAS-B (Fig. 3E). In both cell lines, the number of tubules, tubule length, and the number of junctions were all significantly decreased in NECTIN4 siRNA-transfected compared with control cells (Fig. 3F). To confirm that the inhibition of VEGFR2 leads to the decreased cell proliferation and angiogenesis of these cell lines as NECTIN4-knockdown did, cells were treated with cabozantinib, a potent VEGFR2 inhibitor and assessed. Inhibition of VEGFR2 strongly inhibited cell proliferation and angiogenesis in both cell lines, demonstrating the importance of VEGFR2 in angiosarcoma cells (Supplementary Fig. S10). Thus, NECTIN4 may be involved in the regulation of angiogenesis in angiosarcoma cells through regulating VEGFR2 expression.

### Effects of NECTIN4 on EMT in angiosarcoma cells

Because NECTIN4 is known to be involved in EMT^13,23^, we evaluated the expression of EMT-related molecules. Although gene expression of E-cadherin (*CDH1*), zinc finger E-box binding homeobox 1 (*ZEB1*), and zinc finger E-box binding homeobox 2 (*ZEB2*) was slightly but significantly induced in NECTIN4 siRNA-transfected cells, there were no obvious changes in E-cadherin, ZEB1, or ZEB2 expression at the protein level (Supplementary Figs S11, S12). These factors were not changed in NECTIN4 siRNA-transfected ISO-HAS-B (Supplementary Fig. S11).

### Effects of NECTIN4 on Akt and Src signaling

**Figure 4:**
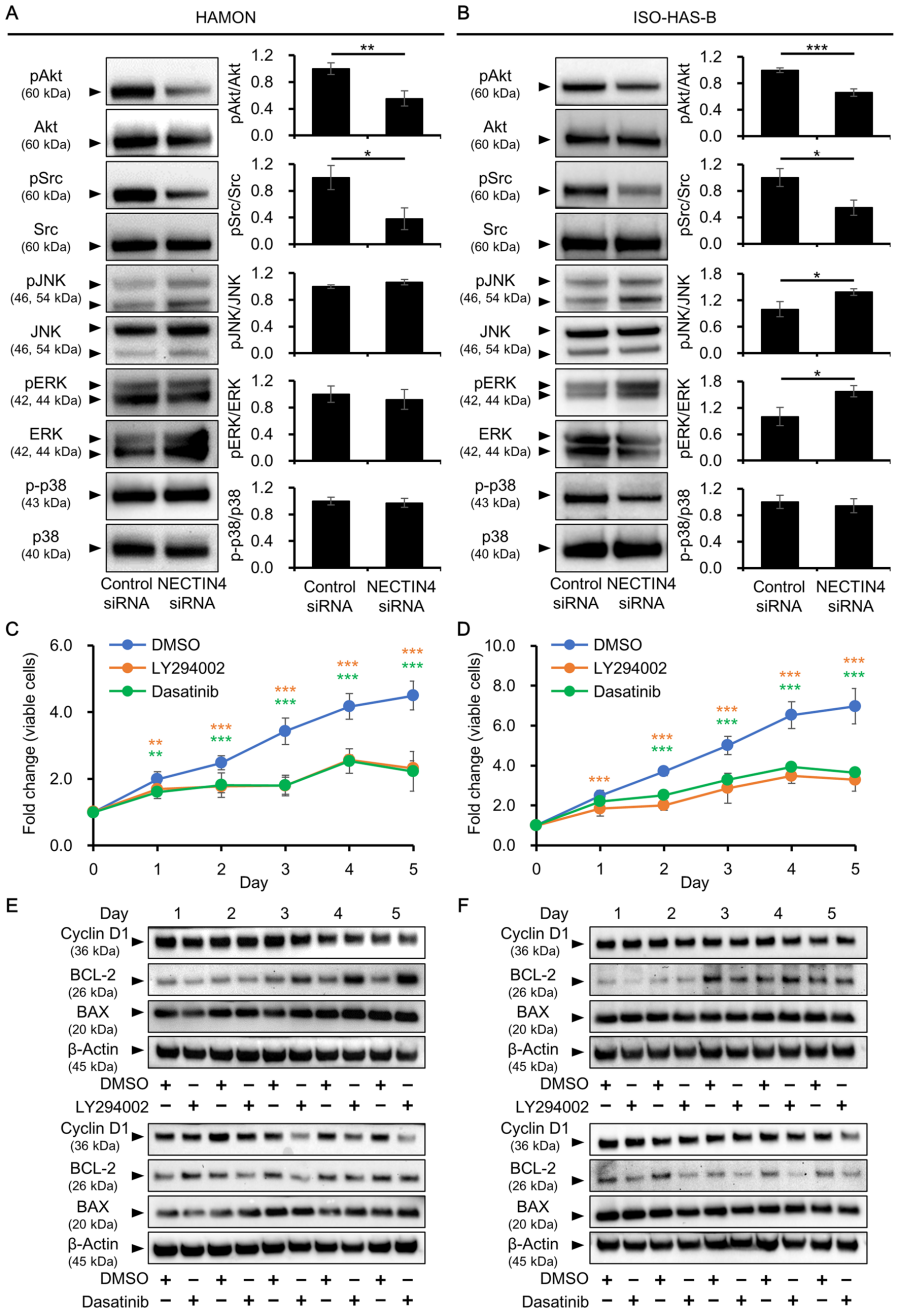
NECTIN4 functions partly through by modulating PI3K/Akt and Src signaling. (**A,B**) Phosphorylation of signaling molecules in control and NECTIN4 siRNA-transfected cells, as determined by western blotting. Representative blot images and ratio of total to phosphorylated (p) proteins in (**A**) HAMON and (**B**) ISO-HAS-B cells. Data are the mean ± SD of three independent experiments. The signal for each phosphorylated protein was analysed using ImageJ software and divided by the signal for the total protein of each molecule. The images shown derive from the same experiment and the blots were processed in parallel. Full-length blots are presented in Supplementary Figs S13 and S14. *P < 0.05 and **P < 0.01 compared with control siRNA-transfected cells. (**C,D**) HAMON and ISO-HAS-B cells were treated with DMSO (0.1%), LY294002 (10 μM), or dasatinib (100 nM) for 1–5 days and assessed for viability. Viable cell number following DMSO-, LY294002-, or dasatinib-treated (**C**) HAMON and (**D**) ISO-HAS-B cells determined using the CCK-8 assay. Data are shown as fold changes relative to Day 0 and are the mean ± SD of three independent experiments, with three wells used for each concentration. ***P < 0.001 compared with DMSO-treated cells. (**E,F**) Cyclin D1, BCL-2, and BAX expression in DMSO-, LY294002-, and dasatinib-treated cells. Representative blot images of (**E**) HAMON and (**F**) ISO-HAS-B cells are shown. The images shown derive from the same experiment and the blots were processed in parallel. Membranes were cut according to the molecular weight to separately hybridize with different antibodies and original blot images are presented in Supplementary Figs S18 and S19.

To elucidate downstream signals of NECTIN4, western blotting was used to evaluate the phosphorylation status of the signaling molecules Akt (downstream of PI3K), proto-oncogene tyrosine protein kinase Src, c-Jun N-terminal kinase (JNK), extracellular signal-regulated kinase (ERK), and p38 mitogen-activated protein kinase. These signaling molecules reportedly regulate biological processes, including proliferation and angiogenesis, in various types of cancer cell^29^. Of the signaling molecules evaluated, there was a significant decrease in the phosphorylation of Akt and Src in NECTIN4 siRNA-transfected HAMON and ISO-HAS-B cells compared with control siRNA-transfected cells. However, NECTIN4 knockdown had no effect on JNK, ERK, or p38 phosphorylation (Fig. 4A,B; Supplementary Figs S13, S14).

### Effects of PI3K/Akt and Src signaling inhibition on angiosarcoma cell proliferation

To verify the involvement of PI3K/Akt and Src signaling in the action of NECTIN4, these signaling pathways were inhibited using specific PI3K and Src inhibitors. HAMON and ISO-HAS-B cells were treated with DMSO (control), LY294002 (PI3K inhibitor), or dasatinib (Src inhibitor), and the number of viable cells and angiogenesis were assessed. Western blotting was used to evaluate inhibition of Akt or Src phosphorylation. Akt phosphorylation was significantly inhibited 1–4 days after treatment with LY294002, but gradually recovered in HAMON (Supplementary Figs S15, S16). In ISO-HAS-B, LY294002 significantly inhibited Akt phosphorylation 1–5 days after treatment (Supplementary Figs S15, S16). Src phosphorylation was strongly and significantly inhibited by dasatinib 1–5 days after treatment in both cell lines (Supplementary Figs S15, S16). Treatment of HAMON and ISO-HAS-B cells with either LY294002 or dasatinib significantly decreased the number of viable cells compared with the DMSO-treated control (Fig. 4C,D).

We also determined the effects of LY294002 and dasatinib on cyclin D1, BCL-2, and BAX expression. In LY294002-treated HAMON cells, BCL-2 was significantly upregulated compared with DMSO-treated cells. LY294002 had no significant effect on protein levels of Cyclin D1 and BAX (Fig. 4E; Supplementary Figs S17, S18). In dasatinib-treated HAMON cells, cyclin D1 expression was significantly downregulated compared with the DMSO-treated control. Dasatinib treatment downregulated BCL-2 and slightly but significantly upregulated BAX (Fig. 4E; Supplementary Fig. S17). LY294002 had no effect on cyclin D1, BCL-2, or BAX in ISO-HAS-B cells (Fig. 4F; Supplementary Figs S17, S19). Treatment with dasatinib significantly downregulated BCL-2 in ISO-HAS-B, but did not change BAX (Fig. 4F; Supplementary Fig. S17, S19).

### Angiogenesis of angiosarcaoma cells after Src and PI3K/Akt signaling inhibition

**Figure 5:**
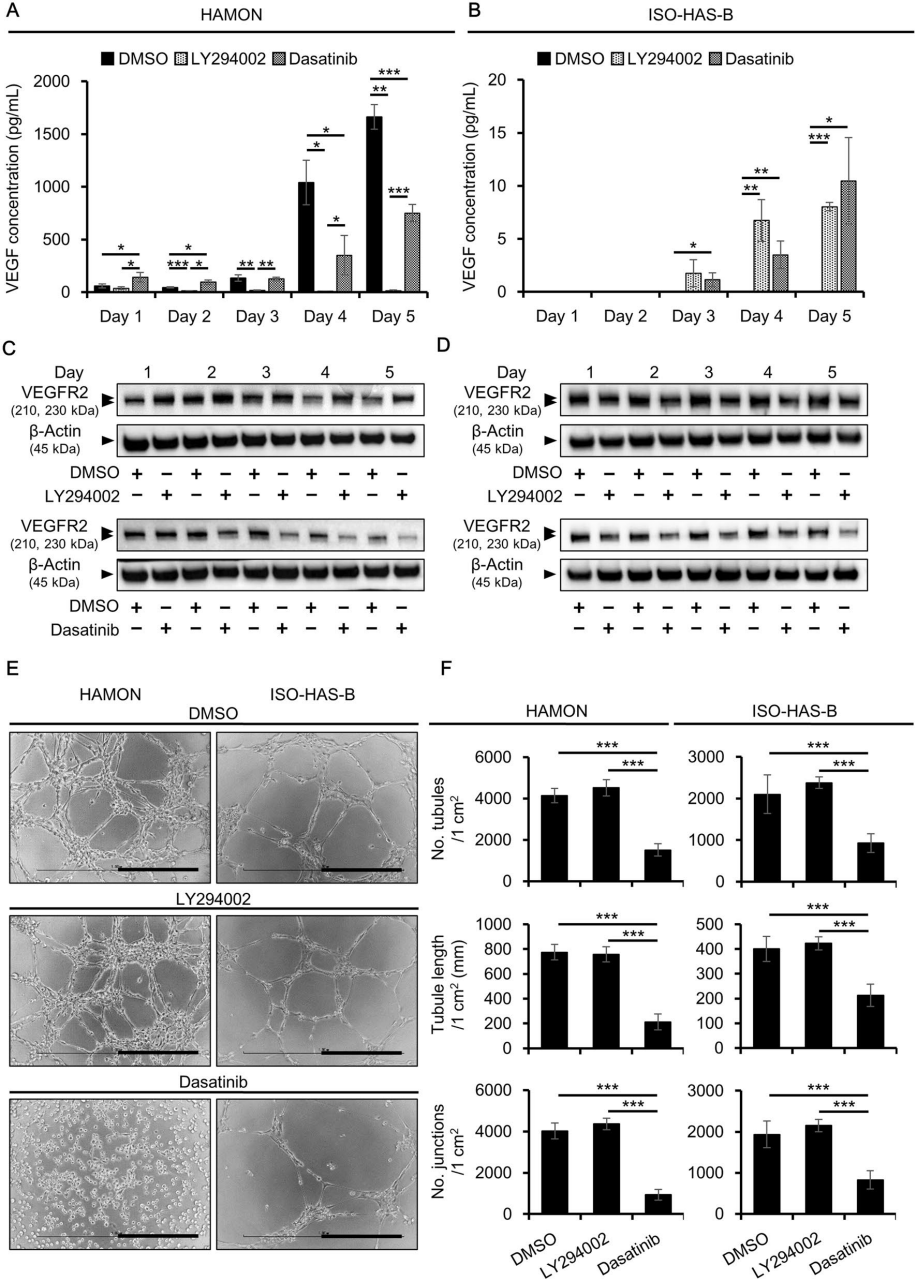
Inhibition of Src prevents angiogenesis in angiosarcoma cells. HAMON and ISO-HAS-B cells were treated with DMSO (0.1%), LY294002 (10 μM), or dasatinib (100 nM) for 1–5 days and assessed for angiogenesis. (**A,B**) VEGF protein concentration in the culture supernatant of (**A**) HAMON and (**B**) ISO-HAS-B cells. Data are the mean ± SD of three independent experiments. (**C,D**) Representative blot images of VEGFR2 and β-actin. The VEGFR2 signal was analysed using ImageJ software and was normalized against that of β-actin. The images shown derive from the same experiment and the blots were processed in parallel. Membranes were cut according to the molecular weight to separately hybridize with different antibodies and original blot images are presented in Supplementary Fig. S21. (**E**) Representative images of angiogenesis. Scale bars = 0.5 mm. (**F**) Number of tubules per 1 cm^2^, tubule length per 1 cm^2^, and the number of junctions per 1 cm^2^ of DMSO-, LY294002-, and dasatinib-treated HAMON (left) and ISO-HAS-B (right) cells in the angiogenesis assay. Data are the mean ± SD of three independent experiments. *P < 0.05, **P < 0.01, and ***P < 0.001.

We further assessed the effects of the PI3K and Src inhibitors on angiogenesis. In HAMON cells the production of VEGF was significantly reduced in LY294002- compared with DMSO-treated cells over 1–5 days of treatment. VEGF secretion was significantly induced in dasatinib- compared with DMSO-treated cells at 1 and 2 days after treatment, but was significantly lower in the dasatinib-treated cells on Days 4 and 5 (Fig. 5A). In ISO-HAS-B cells, VEGF was significantly induced in LY294002-treated condition on Days 4 and 5 and dasatinib-treated condition on Days 3–5 (Fig. 5B). We also assessed VEGFR2 expression. LY294002 significantly upregulated VEGFR2 protein on Day 4 in HAMON cells. Conversely, dasatinib significantly downregulated VEGFR2 expression especially on Days 3–5 of treatment (Fig. 5C; Supplementary Figs S20, S21). LY294002 had no effect on VEGFR2 in ISO-HAS-B (Fig. 5D; Supplementary Figs S20, S21). Similar to HAMON cells, dasatinib significantly downregulated VEGFR2 expression especially on Days 2–5 of treatment (Fig. 5D; Supplementary Figs S20, S21).

Angiogenesis was also investigated in cells treated with DMSO, LY294002, or dasatinib. DMSO-treated cells formed tubular structures on ECM gels, and this tube formation was strongly inhibited by dasatinib in both cell lines, but not LY294002 (Fig. 5E). The number of tubules, tubule length, and the number of junctions were all significantly decreased in dasatinib-treated cells compared with DMSO-treated control cells (Fig. 5F).

### Effects of Src signaling reactivation in NECTIN4-knockdown angiosarcoma cells.

**Figure 6:**
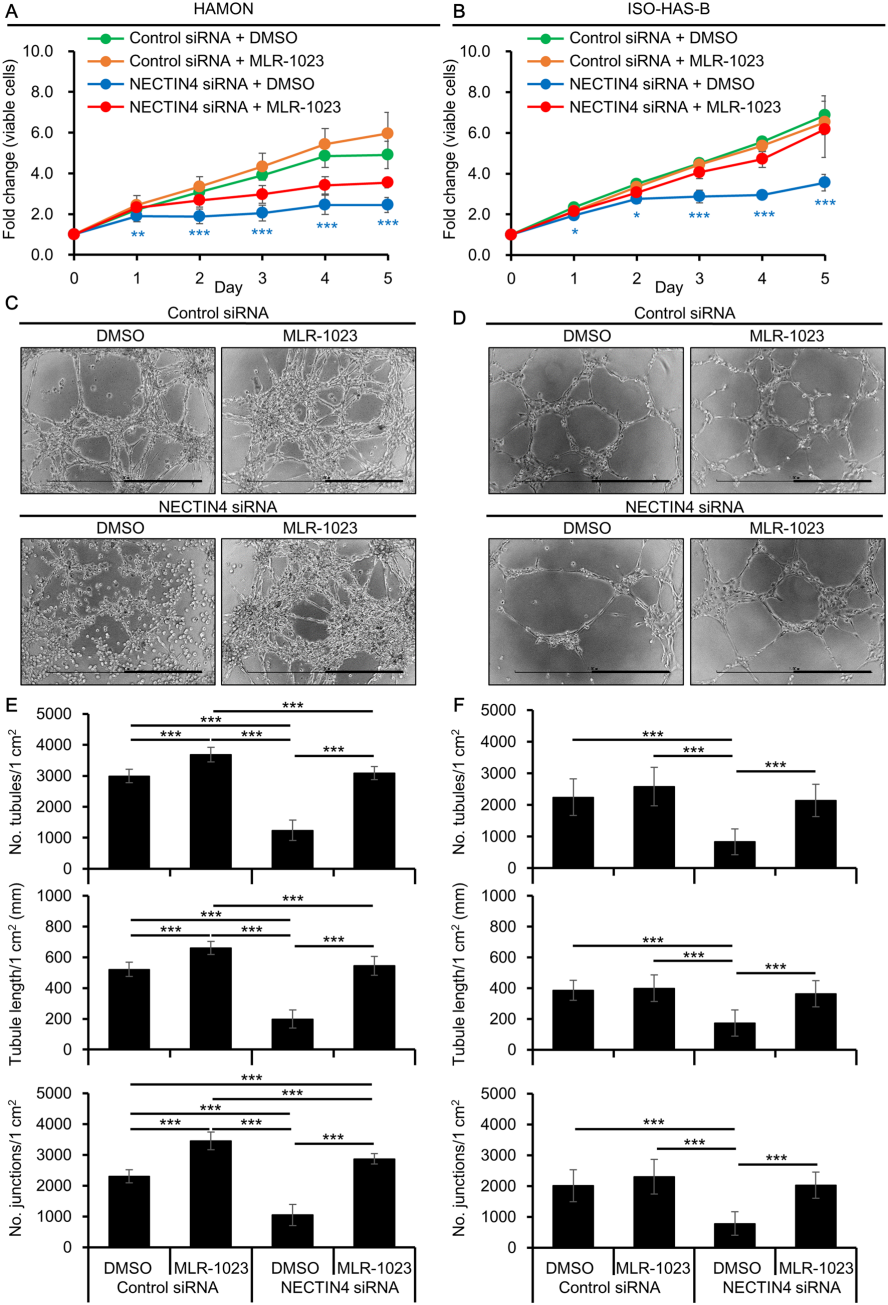
Reactivation of Src signaling in NECTIN4-inhibited angiosarcoma cells. Control or NECTIN4 siRNA-transfected HAMON and ISO-HAS-B cells were further treated with MLR-1023 and assessed for cell proliferation and angiogenesis. (**A,B**) Mean (± SD) number of viable cells in siRNA-transfected, DMSO (0.1%) or MLR-1023 (1 μM)-treated (**A**) HAMON and (**B**) ISO-HAS-B cells, as detected by the CCK-8 assay. Data show fold changes relative to Day 0. Experiments were repeated three times, with three wells used for each condition. Significance of differences was calculated between NECTIN4-siRNA transfected DMSO-treated cells and NECTIN4-siRNA transfected MLR-1023-treated cells. (**C,D**) Representative images of angiogenesis in (**C**) HAMON and (**D**) ISO-HAS-B cells. Scale bars = 0.5 mm. (**E,F**) Number of tubules per 1 cm^2^, tubule length per 1 cm^2^, and the number of junctions per 1 cm^2^ of (**E**) HAMON and (**F**) ISO-HAS-B cells in the angiogenesis assay. Data are the mean ± SD of three independent experiments. **P < 0.01, and ***P < 0.001.

To further investigate the involvement of Src signaling in the action of NECTIN4, Src was reactivated in the NECTIN4 siRNA-transfected cells and cell proliferation and angiogenesis was assessed. MLR-1023 was used as an activator, which is reported to induce phosphorylation of Src^30^. When NECTIN4 siRNA-transfected cells were further treated with MLR-1023, the number of live cells was significantly increased compared with NECTIN4 siRNA-transfected, DMSO-treated cells in both HAMON and ISO-HAS-B (Fig. 6A,B). In the angiogenesis assay, treatment with MLR-1023 restored the formation of tubular structures which was inhibited by NECTIN4 knockdown in HAMON and ISO-HAS-B cells (Fig. 6C,D). The number of tubules, tubule length, and the number of junctions were all increased by MLR-1023 treatment in NECTIN4 siRNA-transfected HAMON and ISO-HAS-B cells (Fig. 6E,F). We also tested effects of dasatinib in NECTIN4-knockdown cells. Simultaneous treatment with NECTIN4 siRNA and dasatinib did not further decrease the number viable cells in angiosarcoma cell lines (Supplementary Fig. S22). Angiogenesis was strongly inhibited in NECTIN4 siRNA-transfected dasatinib-treated condition compared to NECTIN4 siRNA-transfected DMSO-treated condition in HAMON cells, but not in ISO-HAS-B (Supplementary Fig. S22).

Together, these results imply that NECTIN4 affects the number of viable cells and angiogenesis of angiosarcoma cells partly by controlling Src signaling.

## Discussion

The development of effective treatments for angiosarcoma is a long-standing challenge, especially for patients with advanced, metastasized, and/or unresectable lesions. Although various approaches have been tried, treatment efficacy remains unsatisfactory and more effective treatments are needed. In this study we focused on NECTIN4 as a potential target of angiosarcoma therapy, and report, for the first time, that NECTIN4 is expressed in angiosarcoma lesions of patients. NECTIN4 was highly expressed in angiosarcoma cells compared with normal endothelial cells. Knockdown of NECTIN4 attenuated cell survival and angiogenesis of human angiosarcoma cells, emphasizing the potential of NECTIN4 as a therapeutic target.

We found varied expressions of NECTIN4 in patients’ samples. When we analyzed the correlation between NECTIN4 expression and clinical factors, NECTIN4 was frequently positive in tumors with high-grade malignant potential (i.e. non-skin angiosarcoma or epithelioid histopathological subtype), although the correlation was statistically not significant. Because the number of these high-grade samples is still small, continuous assessment of NECTIN4 in these angiosarcoma samples is needed to further evaluate the significance of NECTIN4 in angiosarcoma.

Knockdown of NECTIN4 altered the expression of cell proliferation- and cell survival-related molecules (i.e. cyclin D1, BCL-2, and BAX), resulting in a decrease in the number of viable cells and in an increase in the apoptotic cells. Cyclin D1 is a member of the cyclin family, which regulates cell proliferation by controlling transition from the G_1_ to S phase of the cell cycle. Cyclin D1 is overexpressed in many types of tumors, including angiosarcoma^31–33^. Although little is known about the relationship between NECTIN4 and cell cycle, it was reported that inhibition of NECTIN4 resulted in the increase of sub-G1 cell in 5-FU treated colon cancer cells^34^. Using cell cycle analysis we also found that knockdown of NECTIN4 alter the proportion of G0/G1 and S phase in angiosarcoma cells, i.e. G0/G1 non-proliferating cells were increased in NECTIN4 siRNA-transfected condition (Supplementary Fig. S8). Thus, it is indicated that NECTIN4 might be involved in the regulation of cell cycle of angiosarcoma cells.

In addition to affecting cyclin D1 expression, NECTIN4 knockdown altered the expression of BCL-2 and BAX. BCL-2 and BAX are well-known anti-apoptotic and apoptotic molecules, respectively, that regulate the apoptosis and survival of cells, including cancer cells^35,36^. Previous studies reported that NECTIN4 overexpression prevents apoptosis and that inhibition of NECTIN4 induces apoptosis, although the detailed mechanisms have not yet been elucidated. For example, in colon cancer cells, NECTIN4-overexpressing cells acquired resistance to 5-fluorouracil, but inhibition of NECTIN4 increased cell sensitivity to 5-fluorouracil and induced apoptosis^34^. NECTIN4 knockdown also induces apoptosis in TPC-1 and KTC-1 papillary thyroid cancer cells *in vitro*^15^. In the present study, NECTIN4 knockdown downregulated BCL-2 and upregulated BAX in angiosarcoma cell lines and induced apoptosis. Because NECTIN4 knockdown also decreased Akt and Src phosphorylation, we speculate that these signals are involved in regulation of BCL-2 and/or BAX by NECTIN4 and affects tumor progression of angiosarcoma. It is reported that inhibition of PI3K/mTOR signaling pathway suppress growth of angiosarcoma in vivo using mouse model^37^. In another study, inhibition of Shb, which interacts with Src and regulate its downstream signaling, also suppress angiosarcoma in in vivo xenograft model^38^. However, treatment of HAMON cells with LY294002 (PI3K/Akt inhibitor) and dasatinib (Src inhibitor) did not exactly reproduce the BCL-2 and BAX expression patterns seen after NECTIN4 knockdown. Thus, other signals may be responsible for the regulation of BCL-2 and BAX by NECTIN4. At present, little is known about how NECTIN4 regulates these molecules and apoptosis, and these issues will be addressed in future research.

Angiogenesis is an important process of tumor progression in various cancers, including angiosarcoma^26,27^. In particular, the VEGFA (including VEGF_165_)–VEGFR2 axis plays a central role in angiogenesis in endothelial cells^39,40^. NECTIN4 expression was reported to be positively correlated with VEGF expression in pancreatic cancer^26^, but we found that VEGF expression was induced by NECTIN4 knockdown in angiosarcoma cells. In contrast to VEGF expression, the expression of VEGFR2, a VEGF receptor, was significantly inhibited by NECTIN4 knockdown, and eventually angiogenesis itself was inhibited. Thus, we speculate that VEGF expression was upregulated following knockdown of NECTIN4 to compensate for the downregulation in VEGFR2, but it failed to promote angiogenesis because it could not transduce its signal through VEGFR2. It is unknown how NECTIN4 affects VEGFR2 expression, but we found that Src inhibition suppressed VEGFR2 expression in angiosarcoma cells, as seen after NECTIN4 knockdown. In addition, reactivation of Src partly canceled the effect of NECTIN4 knockdown. Previous studies indicate that NECTIN4 regulates Src signaling in human epithelial cells. For example, NECTIN4 maintained survival of human mammary epithelial cell lines by activating integrin α_6_β_4_/protein tyrosine phosphatase non-receptor type 11/c-Src signaling^41,42^. It has also been reported that an interaction between breast cancer cell-derived NECTIN4 ecto-domain and integrin β_4_ promotes angiogenesis in HUVEC via Src signaling^43^. These observations suggest that NECTIN4 regulates VEGFR2 expression and angiogenesis in part by modulating Src signaling.

The NECTIN4-targetting ADC enfortumab vedotin is gaining attention in the treatment of several types of malignant tumors, such as advanced urothelial cancer^17,18^ and several solid tumors^19^. However, the efficacy of enfortumab vedotin in the treatment of skin tumors has not been examined in detail. Based on the results of the present study, we suggest NECTIN4 may be a promising therapeutic target of angiosarcoma because it will support drug recognition of angiosarcoma cells. Taking the results of this study into consideration, targeting NECTIN4 has further advantages beyond serving as a landmark for drug recognition:

1. NECTIN4 expression was significantly higher in angiosarcoma cells than normal endothelial cells. Thus, NECTIN4-targetting ADC may have a greater effect on angiosarcoma cells than on normal endothelial cells, decreasing adverse effects. Of note, normal endothelial cells were less sensitive to MMAE, the cytotoxic part of enfortumab vedotin; this implies that adverse effects on normal cells may be further avoided by careful selection of the dose of ADC.
2. NECTIN4 knockdown decreased the number of viable angiosarcoma cells and induced apoptosis; thus, NECTIN4 inhibition may contribute to the suppression of the growth of tumor cells.
3. NECTIN4 inhibition of strongly inhibited angiogenesis, likely through downregulation of VEGFR2.

Anti-angiogenic therapy has recently attracted attention as a novel therapeutic strategy^9,10^; thus, targeting NECTIN4 may also contribute to tumor suppression by preventing angiogenesis. The limitation of our results is that NECTIN4-targeted ADC such as enfortumab vedotin is not available for us at present and we could not evaluate in vivo therapeutic significance of NECTIN4-targted ADC using a xenograft animal model. It will be a future challenge to assess the potential of NECTIN4-targeted ADC in vivo to reinforce our findings.

In conclusion, we have revealed that NECTIN4 is expressed in human angiosarcoma tissues. In addition, NECTIN4 was involved in regulating the proliferation, survival, and angiogenesis of angiosarcoma cells, partly through Src signaling. We also suggest that NECTIN4-targeted therapy may be a promising novel treatment for angiosarcoma.

## Methods

### Patients

A retrospective review was performed in accordance with the guidelines of the Declaration of Helsinki. Immunohistochemical analysis of patients’ samples was approved by the Ethics Committee of Kyushu University Hospital (Approval no. 30-363, November 27, 2018). Written informed consent was obtained from patients prior to their inclusion in the study. Seventy-four patients with angiosarcoma lesions who had been treated at the Department of Dermatology of Kyushu University, Fukuoka, Japan, between September 1984 and December 2019 were included in the study. At least three experienced dermatopathologists confirmed the diagnosis. Patients’ clinical and demographic data were collected from patient files and analyzed.

### Immunohistochemistry

Formalin-fixed, paraffin-embedded angiosarcoma tissues were obtained from the archives of Kyushu University Hospital. Tissues were sliced into 4-μm sections and stained as reported previously^23,24^. The sections were incubated with rabbit anti-human NECTIN4 primary antibody (1:150; Abcam, Cambridge, UK; ab192033) for 30 min at room temperature, followed by incubation with N-Histofine Simple Stain AP MULTI secondary antibody (Nichirei Biosciences, Tokyo, Japan; 414261) for 30 min at room temperature. Sections were then treated with the chromogenic substrate FastRed II (Nichirei Biosciences; 415261) and counterstained with hematoxylin (Muto Pure Chemicals, Tokyo, Japan; 30002). NECTIN4 expression was independently assessed by two dermatologists (MM and TI) who were blinded to the patients’ clinical information. The percentage of NECTIN4-positive tumor cells was determined. Sections were observed and images were obtained using a Nikon ECLIPSE 80i microscope (Nikon, Tokyo, Japan).

### Reagents

MMAE (ChemScene, Deerpark, NJ; CS-0837), LY294002 (Abcam; ab120243), dasatinib (Abcam; ab142050), MLR-1023 (Sigma-Aldrich, St. Louis, MO; SML0361), and cabozantinib (Selleck Biotech, Tokyo, Japan; BMS-907351) were dissolved in DMSO (Sigma-Aldrich; 07-4860-5), which was also used as the vehicle control (final concentration 0.1% DMSO).

### Cell culture

HUVEC (Takara Bio, Kusatsu, Japan; C-12203), HDMEC (Takara Bio; C-12212), HDBEC (Takara Bio; C-12211), and HAMON human angiosarcoma cells^44,45^ (kindly provided by Riichiro Abe, Division of Dermatology, Niigata University) were cultured in Endothelial Cell Growth Medium 2 (Takara Bio; C-22111), containing 2% fetal calf serum, 5 ng/mL human epidermal growth factor, 0.2 μg/mL hydrocortisone, 22.5 μg/mL heparin, 20 ng/mL R3 insulin-like growth factor-1, 1 μg/mL ascorbic acid, 10 ng/mL human basic fibroblast growth factor, and 0.5 ng/mL VEGF. ISO-HAS-B human angiosarcoma cells (Resource Center for Biomedical Research, Institute of Development, Aging, and Cancer, Tohoku University) were cultured in DMEM (Sigma Aldrich; D6429) supplemented with 10% FBS. Cells were passaged at confluence using a Detach Kit (Takara Bio; C-41210) according to the manufacturer’s instructions, and the medium was changed every 2–3 days. The CycleavePCR Mycoplasma Detection Kit (Takara Bio; CY232) was used to test for mycoplasma contamination, and cells were confirmed to be mycoplasma free. Cell morphology was observed under a microscope and pictures were captured using a microscope camera system (Nikon).

### Immunocytochemistry

Cells were seeded in an eight-well μ-Slide (ibidi, Martinsried, Germany; 80,826) at a density of 1,000 cells/well and incubated for 48 h at 37 °C in 5% CO_2_. Cells were fixed with cold acetone, air-dried, and treated with 5% bovine serum albumin (Sigma-Aldrich; A-2153) to block the non-specific binding of antibodies. After washing with Dulbecco’s phosphate-buffered saline (DPBS, Fujifilm Wako Pure Chemical, Osaka, Japan; 293-72601), fixed cells were incubated with anti-human NECTIN4 primary antibody (1:200; Abcam; ab235897) at 4 °C overnight. After washing with DPBS, cells were incubated with goat anti-rabbit IgG AlexaFluor488 (1:400; Thermo Fisher Scientific, Waltham, MA; A11008) for 30 min at room temperature. Cells were then washed with DPBS and covered with Vectashield mounting medium with 4′,6′-diamidino-2-phenylindole (Vector Laboratories, Burlingame, CA; H-1200) and observed under an EVOS FL fluorescence microscope (Thermo Fisher Scientific).

### siRNA transfection

HAMON and ISO-HAS-B cells were transfected with either negative control siRNA (Invitrogen, Carlsbad, CA; AM4611) or NECTIN4 siRNA (Invitrogen; s37690) using Lipofectamine RNAiMAX (Thermo Fisher Scientific; 13778150) in accordance with the manufacturers’ instructions. Briefly, cells were seeded into six-well plates (2.0 × 10^5^ cells/well), 12-well plates (1.2 × 10^5^ cells/well), or 96-well plates (3,000 cells/well) and incubated for 24 h at 37 °C in 5% CO_2_ before being transfected with the siRNA and incubated for a further 1–5 days at 37 °C in 5% CO_2_. Cell proliferation and in vitro tube formation were then analyzed, as detailed below. NECTIN4 knockdown efficiency was assessed by qRT-PCR and western blotting, and the amount of VEGF secreted into the culture medium was determined by ELISA (see below).

### Cell proliferation assay

The number of viable cells was assessed using a formazan-based assay, namely the Cell Counting Kit-8 (CCK-8; Dojindo, Kumamoto, Japan; 343-07623). Harvested cells were seeded at a density of 3,000 cells/well in 96-well plates and incubated for 24 h at 37 °C in 5% CO_2_. The cells were then used for different experiments to determine the effects of MMAE, NECTIN4 knockdown, the inhibitors LY294002, dasatinib, and cabozantinib, and a Src activator MLR-1023 on cell proliferation. To determine the effects of NECTIN4 knockdown, cells were transfected with siRNA and incubated for 1–5 days, as described above. To assess the effects of LY294002, dasatinib, and cabozantinib, cells were exposed to DMSO (0.1%), LY294002 (10 μM), dasatinib (100 nM), or cabozantinib (10 μM) for 1–5 days at 37 °C in 5% CO_2_. To analyze the effect of dasatinib and MLR-1023 in siRNA-transfected cells, siRNA-transfected cells were exposed to DMSO (0.1%), dasatinib (100 nM), or MLR-1023 (1 μM). The concentrations of the inhibitors/activators used were selected on the basis of previous publications^24,30,46^. CCK-8 solution was then added to each well and the cells were incubated for 3 h at 37 °C. After the incubation, absorbance was measured at 450 nm using a microplate reader (Bio-Rad Laboratories, Hercules, CA).

### IC50 determination

IC50 of MMAE in HUVEC, HDMEC, HDBEC, HAMON, and ISO-HAS-B cells was determined using CCK-8 assay. Harvested cells were seeded at a density of 3,000 cells/well in 96-well plates and incubated for 24 h at 37 °C in 5% CO_2_. The cells were further incubated with fresh medium containing DMSO (0.1%) or various concentrations of MMAE (ranging from 0.1–20.0 nM) for 48 h at 37 °C in 5% CO_2_. CCK-8 solution was then added to each well and the cells were incubated for 3 h at 37 °C. After the incubation, absorbance was measured at 450 nm using a microplate reader (Bio-Rad Laboratories). IC50 was calculated by nonlinear regression analysis of GraphPad Prism 7 software (GraphPad Software, San Diego, CA).

### Cell cycle analysis

Cell cycle of siRNA transfected cells was evaluated using flow cytometry. Briefly, cells were transfected with control or NECTIN4 siRNA as mentioned above and incubated for 5 days at 37 °C in 5% CO_2_. Harvested cells were fixed in 70% ethanol/DPBS and incubated on ice for 30 min. After wash with DPBS, cells were suspended into DPBS containing 2 μg/mL of PI (Invitrogen; P3566) and analyzed using a FACSAriaSORP flow cytometer (BD Biosciences, Franklin Lakes NJ). Cell cycle was then assessed using FlowJo software (Tree Star, San Carlos, CA).

### Annexin V-PI staining

Apoptotic status of siRNA transfected cells were determined by Annexin V-PI staining and flow cytometry analysis. Cells were transfected with control or NECTIN4 siRNA as mentioned above and incubated for 5 days at 37 °C in 5% CO_2_. Harvested cells were then labeled with Annexin V-FITC and PI using the Annexin V-FITC Apoptosis Detection Kit (Nacalai Tesque, Kyoto, Japan; 15,342–54), in accordance with the manufacturer’s instructions. Apoptotic cells were then detected with a FACSAriaSORP flow cytometer and analyzed with FlowJo software.

### In vitro tube formation assay

In vitro tube formation was evaluated using the Angiogenesis Assay Kit (PromoCell, Heidelberg, Germany; PK-CA577-K905) according to the manufacturer’s instructions. Briefly, 50 μL/well ECM solution was added to prechilled 96-well plates and incubated for 1 h at 37 °C to form a gel. HAMON or ISO-HAS-B cells were transfected with control or NECTIN4 siRNA for 48 h, seeded into the ECM-treated wells at a density of 1–2 × 10^4^ cells/well, and incubated for 24 h at 37 °C in 5% CO_2_. To inhibit PI3K/Akt, Src, or VEGFR2, or to activate Src, cells were treated with DMSO (0.1%), LY294002 (10 μM), dasatinib (100 nM), cabozantinib (10 μM), or MLR-1023 (1 μM) respectively for 48 h at 37 °C in 5% CO_2_, seeded into the ECM-treated wells, and then incubated for 24 h at 37 °C in 5% CO_2_. HUVEC were used as a positive control because, in this assay system, they form tubular structures. After incubation, cells were observed under a microscope and images were captured using a microscope camera system (Nikon). The number of tubules, tubule length, and the number of junctions were measured using ImageJ software (National Institutes of Health, Bethesda, MD).

### qRT-PCR

Total RNA was extracted from cells using an RNeasy Mini Kit (Qiagen, Hilden, Germany; 74104) according to the manufacturer’s instructions. RNA was converted to cDNA using a PrimeScript RT Reagent Kit (Takara Bio; RR037), and then subjected to qPCR using SYBR Green Premix Ex Taq (Takara Bio; RR420). The PCR protocol consisted of 95 °C for 30 s, followed by 40 cycles of 95 °C for 5 s and 60 °C for 20 s. The expression of each gene was normalized against the cycle threshold of β-actin (*ACTB*). The primers used for PCR are listed in Supplementary Table S2.

### Western blotting

Cells were seeded in six-well plates at a density of 2 × 10^5^ cells/well, treated with siRNA or inhibitors, and incubated for 1–5 days at 37 °C in 5% CO_2_. After incubation, cells were harvested for protein extraction with M-PER mammalian protein extraction reagent (Thermo Fisher Scientific; 78503) supplemented with protease inhibitor cocktail (Sigma-Aldrich; P8340). The protein concentration of each sample was determined using the BCA assay (Thermo Fisher Scientific; 23227) according to the manufacturer’s instructions. Western blotting was then performed as reported previously^47^. When needed, membranes were cut according to the molecular weight to separately hybridize with different antibodies. Details of the antibodies used for western blotting are summarized in Supplementary Table S3. The resulting immunological bands were reacted with SuperSignal West Pico Chemiluminescent Substrate (Thermo Fisher Scientific; 34580) and images were obtained with the ChemiDoc XRS Plus System (Bio-Rad Laboratories). The intensity of the bands was measured using ImageJ software.

### ELISA

To determine the amount of VEGF secreted from cells, culture supernatants were collected into microcentrifugation tubes and centrifuged at 18,000×*g* for 10 min at 4 °C to remove cell debris. The concentration of VEGF in the culture supernatants was then determined using a Quantikine ELISA Human VEGF kit (R&D Systems, Minneapolis, MN; DVE00) according to the manufacturer’s instructions. As the final step, absorbance was measured at 450 and 570 nm (reference wavelength) using a microplate reader (Bio-Rad Laboratories). Concentrations of VEGF in the samples were calculated based on a standard curve provided as part of the ELISA kit.

### Statistical analysis

Experiments were repeated at least three times. Results are shown as the mean ± SD. Statistical analyses were performed using GraphPad Prism 7. The normality of data distribution was analyzed using the Shapiro–Wilk test. The significance of differences between two groups was assessed using Student’s unpaired two-tailed t-tests. When data were not normally distributed, groups were compared using the Mann–Whitney U test. One-way analysis of variance followed by multiple comparisons was used to assess differences between three or more groups. To assess correlation of NECTIN4 expression and patient’s clinical factors, t-test and Fisher’s exact test was used. *P* < 0.05 was considered statistically significant.

## Supplementary Information

Supplementary Information.

## Data Availability

All data obtained or analyzed during this study are included in the main text and in the supplementary figures/tables.
