# Supplementary material for: Nectin cell adhesion molecule 4 regulates angiogenesis through Src signaling and serves as a novel therapeutic target in angiosarcoma
Source: Sci Rep. 2022 Mar 7;12:4031. doi: 10.1038/s41598-022-07727-x (PMC8901754; doi:10.1038/s41598-022-07727-x)
Supplement: Supplementary file 1 — Supplementary Information. [file 41598_2022_7727_MOESM1_ESM.pdf]

Nectin cell adhesion molecule 4 regulates angiogenesis through Src signaling and serves as a novel therapeutic target in angiosarcoma

Yuka Tanaka[1], Maho Murata[1], Keiko Tanegashima[1], Yoshinao Oda[2], and Takamichi Ito[1, *]

[1]Department of Dermatology, Graduate School of Medical Sciences, Kyushu University, Fukuoka, Japan
[2]Department of Anatomic Pathology, Graduate School of Medical Sciences, Kyushu University, Fukuoka, Japan

*Corresponding author: Takamichi Ito, Department of Dermatology, Graduate School of Medical Sciences, Kyushu University, 3-1-1 Maidashi, Higashi-ku, Fukuoka City, Fukuoka 812-8582, Japan.
Tel.: +81-92-642-5585, Fax: +81-92-642-5600
E-mail: takamiti@dermatol.med.kyushu-u.ac.jp

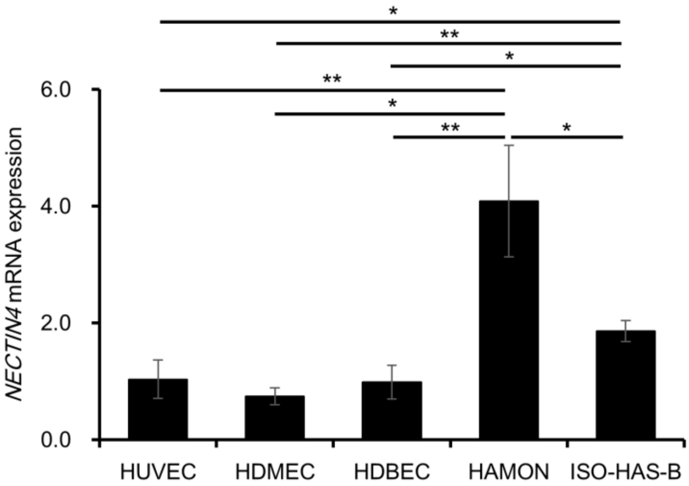

Supplementary Figure S1. ***NECTIN4*** **mRNA expression in normal endothelial cells and in angiosarcoma cells.** Gene expression of *NECTIN4* was measured in HUVEC, HDMEC, HDBEC, HAMON, and ISO-HAS-B cells. Data are the mean ± SD of three independent experiments. **P* < 0.05 and ***P* < 0.01.

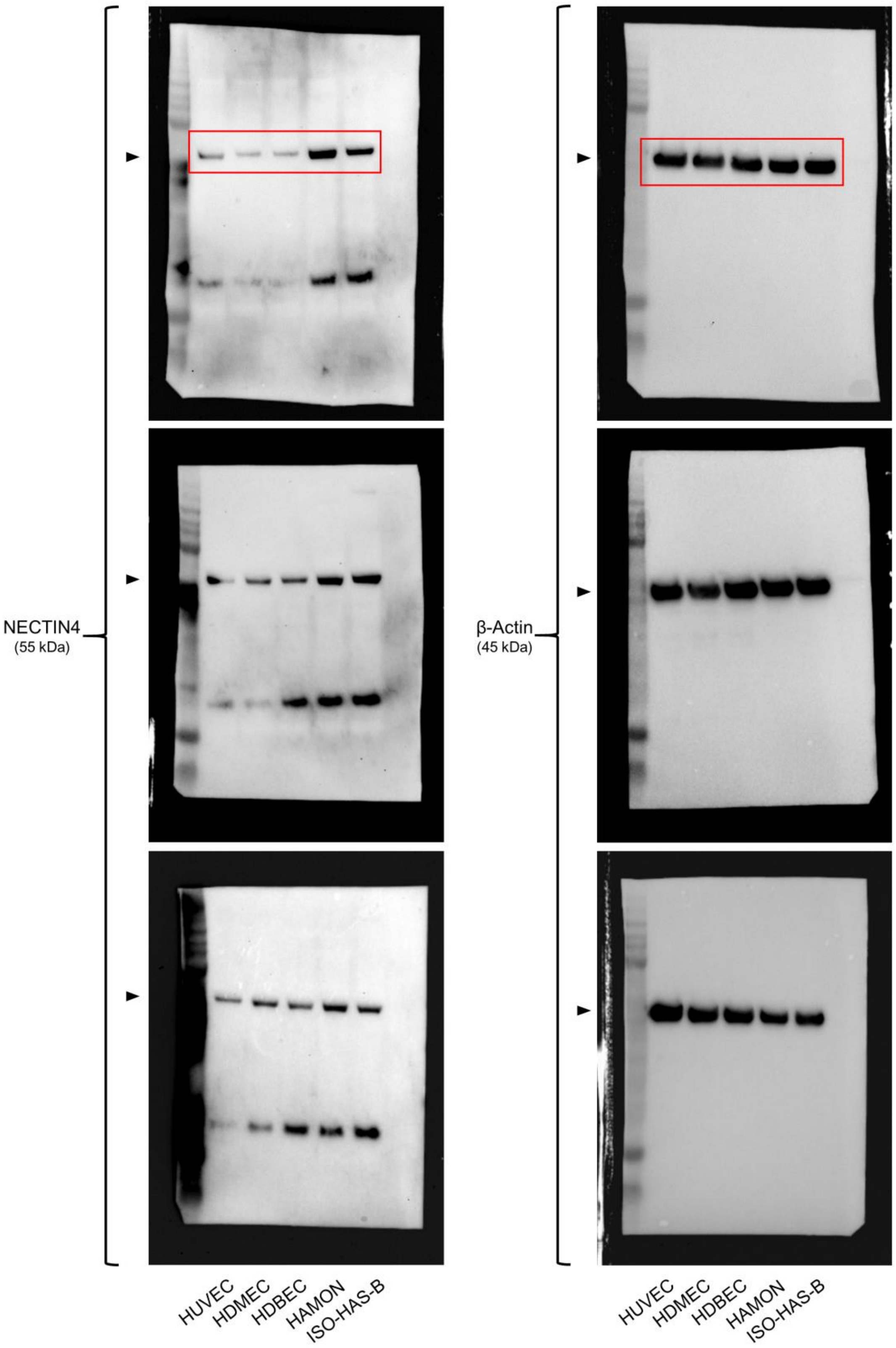

Supplementary Figure S2. Full-length blots presented in Fig. 1C. NECTIN4 and β-actin protein expression in HUVEC, HDMEC, HDBEC, HAMON, and ISO-HAS-B cells was determined by western blotting. Unedited original images of blots are shown. The NECTIN4 signal was analysed using ImageJ software and was normalized against that of β-actin. The images shown derive from the triplicate experiments. The red boxes indicate the croped areas shown in Fig. 1C.

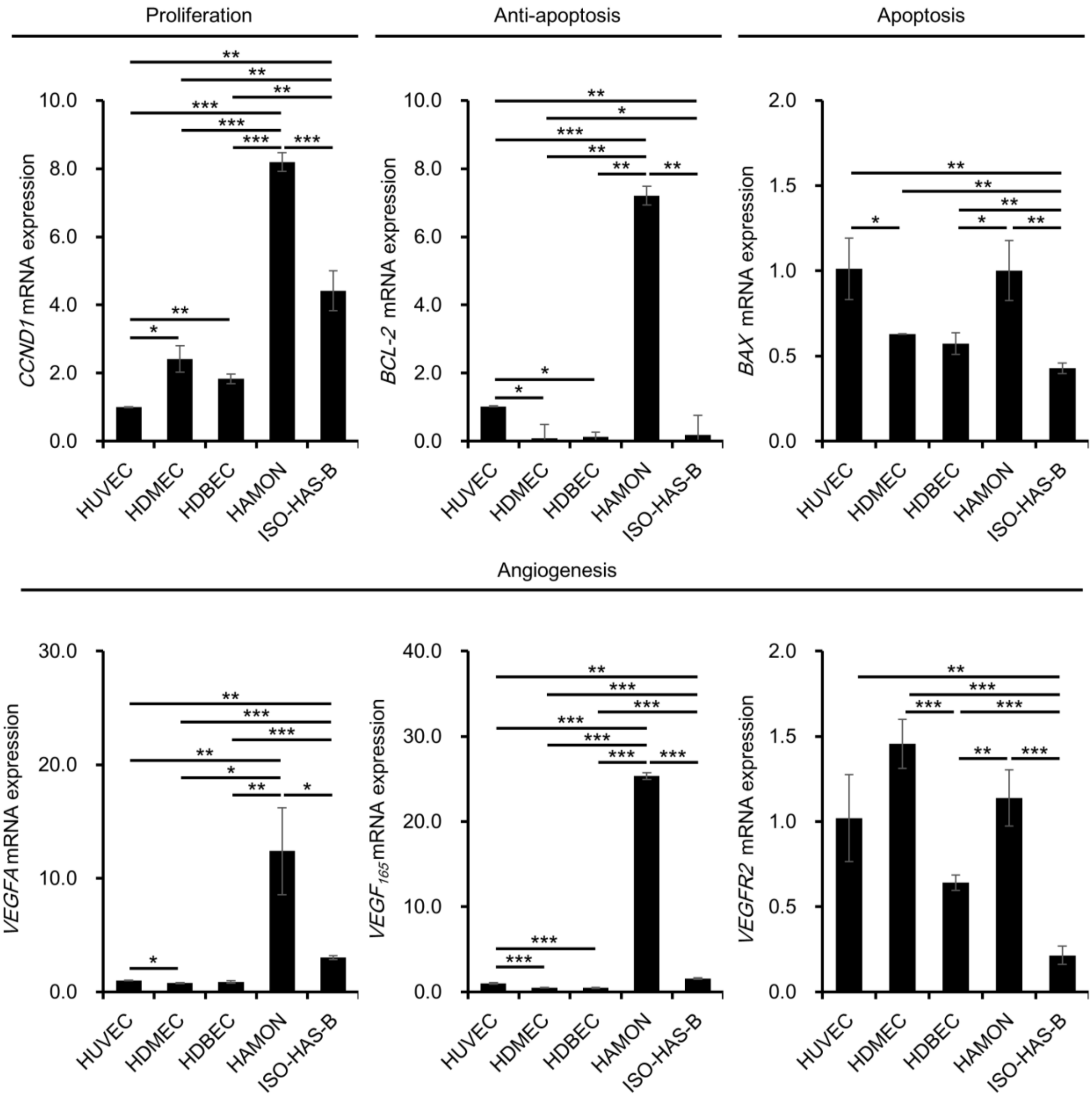

Supplementary Figure S3. Gene expression in normal endothelial cells and in angiosarcoma cells. Gene expression of cyclin D1 (*CCND1*; a cell proliferation marker), *BCL-2* (an anti-apoptotic factor), *BAX* (an inducer of apoptosis), *VEGFA* and $VEGF_{165}$ (potent angiogenic factors), and *VEGFR2* (a VEGF receptor), was measured in HUVEC, HDMEC, HDBEC, HAMON, and ISO-HAS-B cells. Data are the mean ± SD of three independent experiments. *$P < 0.05$, **$P < 0.01$, and ***$P < 0.001$.

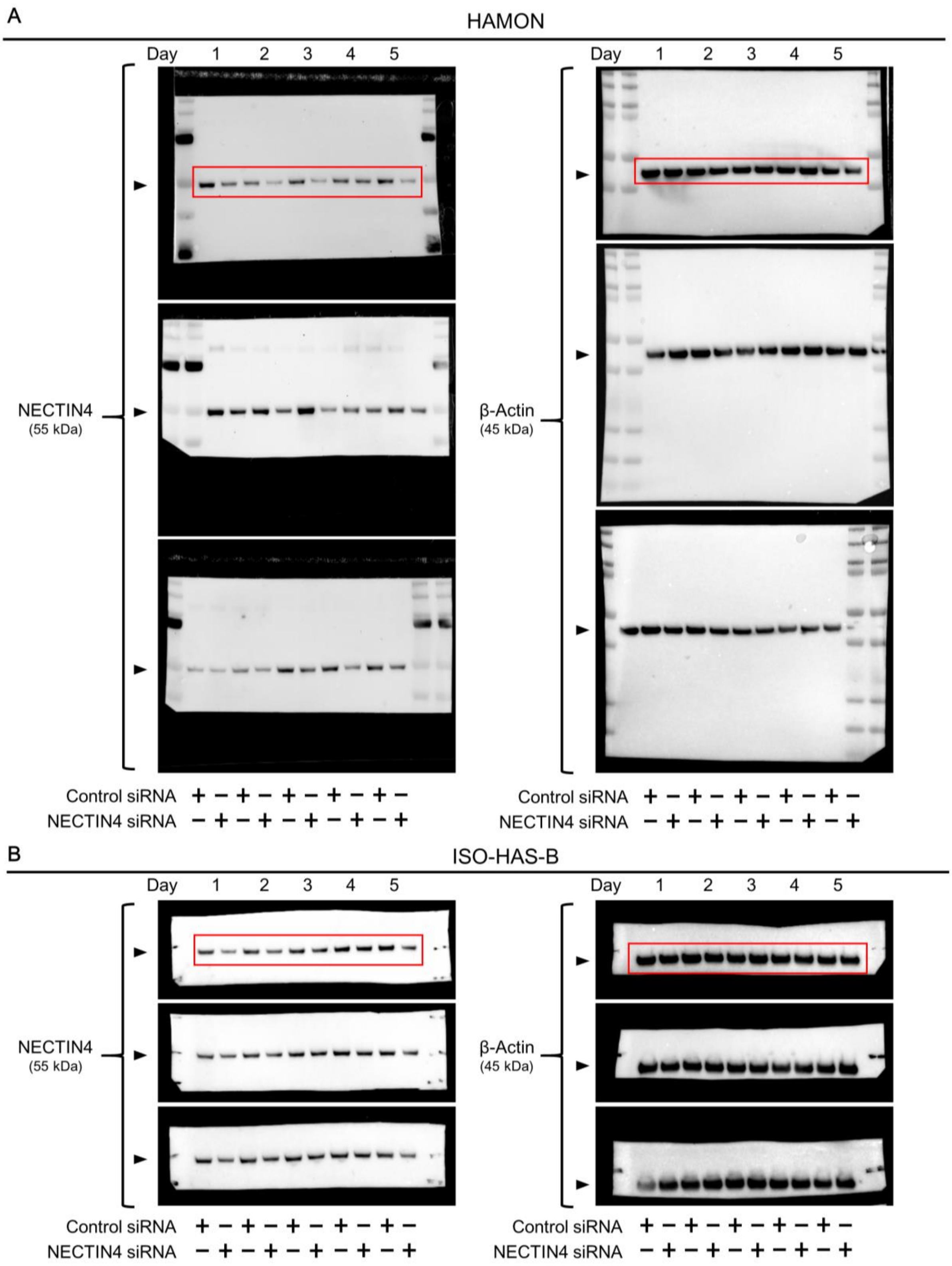

Supplementary Figure S4. Full-length blots presented in Fig. 2A and 2B. Protein expression of NECTIN4 and β-actin in (**A**) HAMON cells and in (**B**) ISO-HAS-B was determined by western blotting. Unedited original images of blots are shown. The signal for each protein was analysed using ImageJ software and was normalized against that of β-actin. The images shown derive from the triplicate experiments. Membranes were cut based on the size marker and hybridized with different kinds of antibodies when needed. The red boxes indicate the croped areas shown in Fig. 2A and 2B.

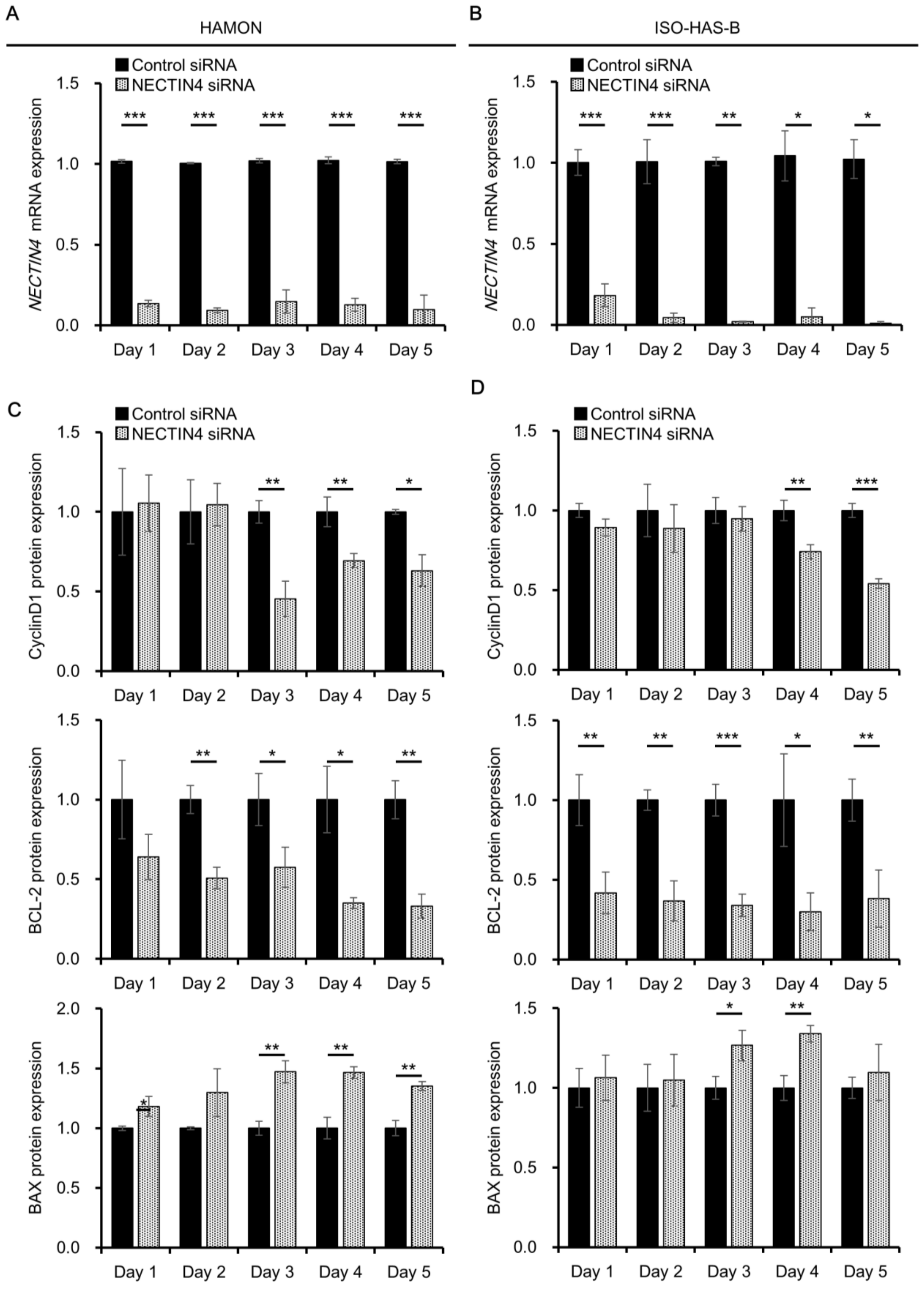

Supplementary Figure S5. Effect of NECTIN4-kncockdown in angiosarcoma cells. HAMON and ISO-HAS-B angiosarcoma cells were transfected with control or NECTIN4 siRNA. (**A**,**B**) Mean (± SD) NECTIN4 knockdown efficiency at the mRNA level in (**A**) HAMON and in (**B**) ISO-HAS-B determined in

three independent experiments. (**C**,**D**) Protein expressions of cyclin D1, BCL-2, and BAX in NECTIN4-knockdown angiosarcoma cell lines. Mean (± SD) cyclin D1, BCL-2, and BAX expressions at the protein levels in (**C**) HAMON and in (**D**) ISO-HAS-B determined in three independent experiments. $^{*}P < 0.05$, $^{**}P < 0.01$, and $^{***}P < 0.001$.

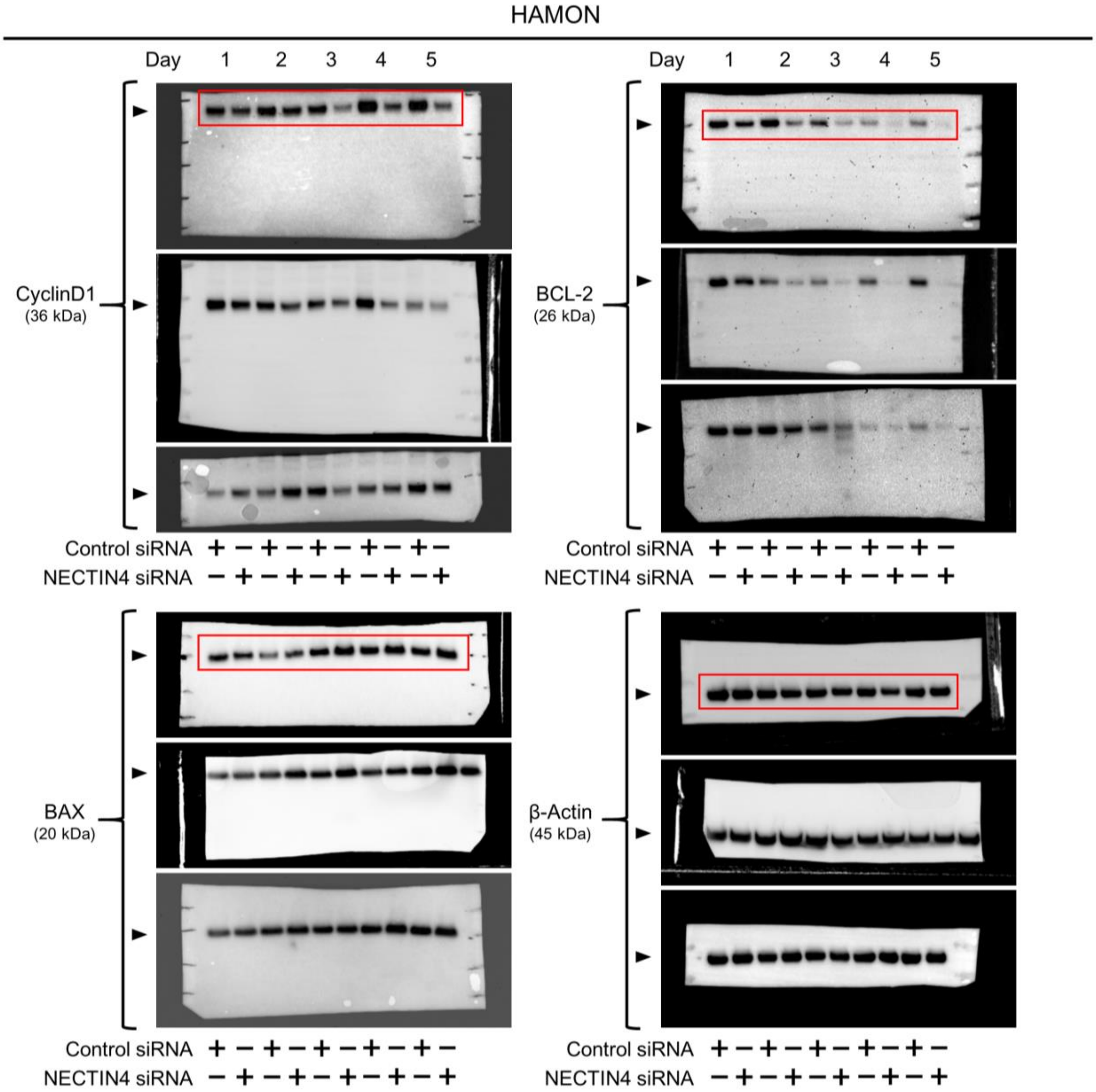

Supplementary Figure S6. Full-length blots presented in Fig. 2E. Protein expression of cyclin D1, BCL-2, BAX, and β-actin in control or NECTIN4 siRNA-transfected HAMON cells was determined by western blotting. Unedited original images of blots are shown. The signal for each protein was analysed using ImageJ software and was normalized against that of β-actin. The images shown derive from the triplicate experiments. Membranes were cut based on the size marker and hybridized with different kinds of antibodies when needed. The red boxes indicate the croped areas shown in Fig. 2E.

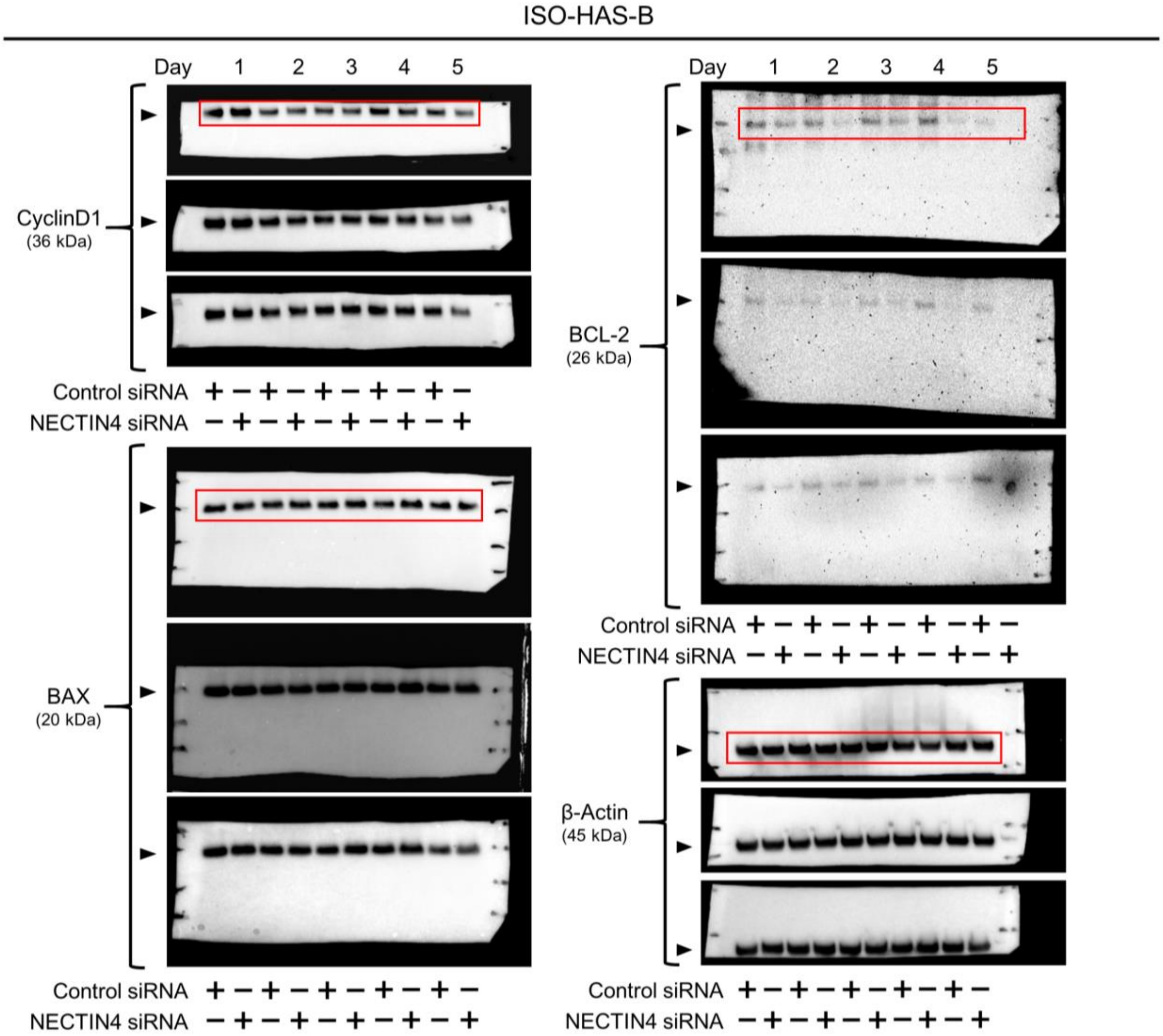

Supplementary Figure S7. Full-length blots presented in Fig. 2F. Protein expression of cyclin D1, BCL-2, BAX, and β-actin in control or NECTIN4 siRNA-transfected ISO-HAS-B cells was determined by western blotting. Unedited original images of blots are shown. The signal for each protein was analysed using ImageJ software and was normalized against that of β-actin. The images shown derive from the triplicate experiments. Membranes were cut based on the size marker and hybridized with different kinds of antibodies when needed. The red boxes indicate the croped areas shown in Fig. 2F.

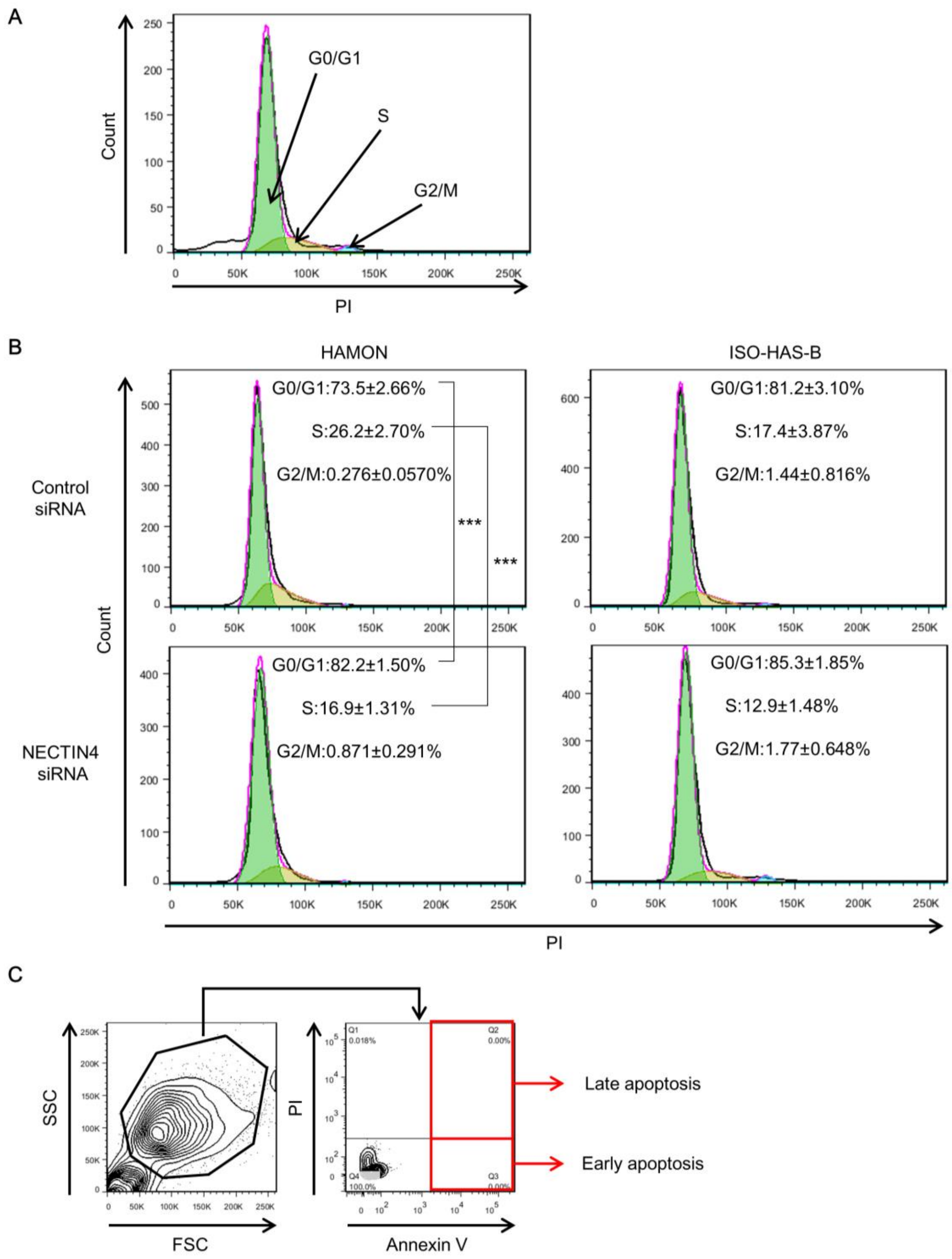

Supplementary Figure S8. Analysis of cell cycle and apoptosis in NECTIN4-knockdown angiosarcoma cells. HAMON and ISO-HAS-B cells were transfected with control or NECTIN4-siRNA and cell cycle and apoptosis were analyzed by flow cytometer. (**A**) Cell cycle was analyzed using PI staining. A representative image of cell cycle analysis showing G0/G1, S, and G2/M phase. (**B**) Representative images of cell cycle analysis in HAMON (left) and in ISO-HAS-B (right) cells. Percentages of each phase were calculated from the results of three independent experiments. ***P < 0.001. (**C**) Gate setting of apoptosis analysis. Cell debris located in low forward scatter (FSC) and low side scatter (SSC) were excluded from the first gate. In the second gate, Annexin V-positive PI-negative cells were defined as early apoptotic cells and Annexin V-positive PI-positive cells were defined as late apoptotic cells.

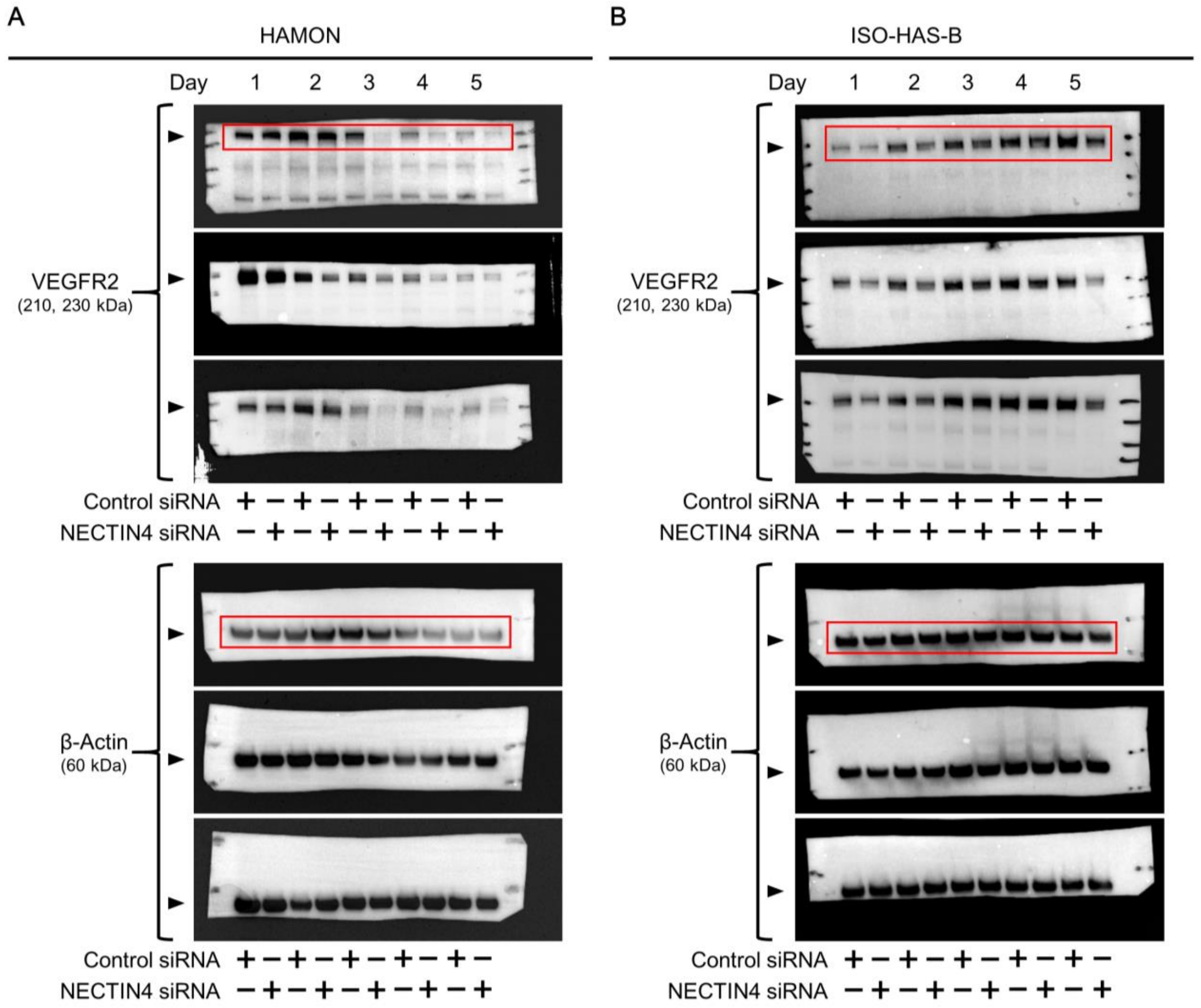

Supplementary Figure S9. Full-length blots presented in Fig. 3C and 3D. VEGFR2 and β-actin protein expression in control or NECTIN4 siRNA-transfected (**A**) HAMON and (**B**) ISO-HAS-B cells was determined by western blotting. Unedited original images of blots are shown. The VEGFR2 signal was analysed using ImageJ software and was normalized against that of β-actin. The images shown derive from the triplicate experiments. Membranes were cut based on the size marker and hybridized with different kinds of antibodies when needed. The red boxes indicate the croped areas shown in Fig. 3C and 3D.

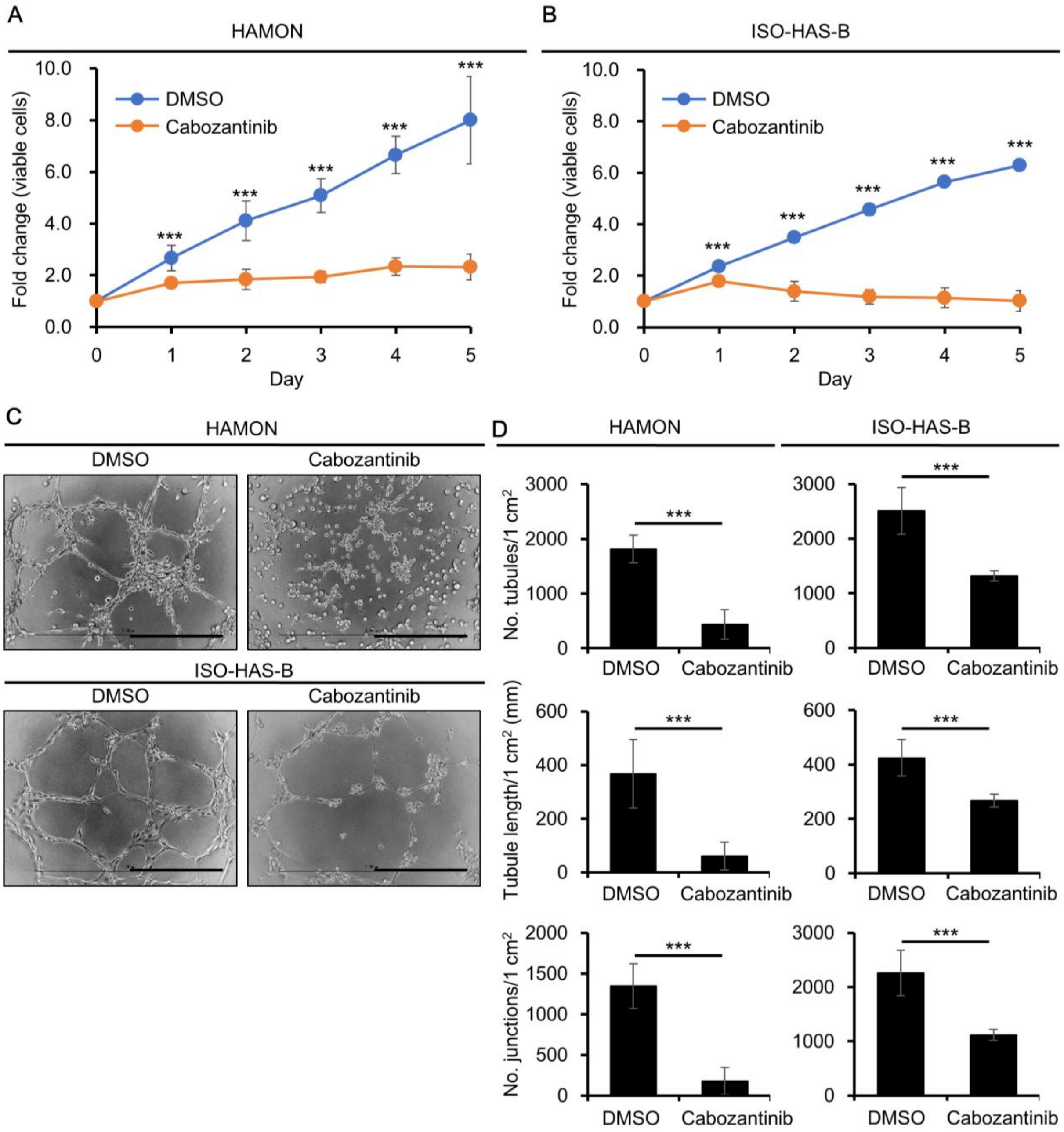

Supplementary Figure S10. Effects of VEGFR2 inhibition on cell proliferation and angiogenesis in angiosarcoma cells. HAMON and ISO-HAS-B cells were treated with DMSO (0.1%) or a potent VEGFR2 inhibitor cabozantinib (10 μM) and assessed for cell proliferation and angiogenesis. (**A,B**) Mean (± SD) number of viable cells in DMSO or cabozantinib-treated (**A**) HAMON and (**B**) ISO-HAS-B cells, as detected by the CCK-8 assay. Data show fold changes relative to Day 0. Experiments were repeated three times, with three wells used for each condition. (**C**) Representative images of angiogenesis assay. Scale bar = 0.5 mm. (**D**) Number of tubules per 1 cm^2, tubule length per 1 cm^2, and the number of junctions per 1 cm^2 for DMSO and cabozantinib-treated HAMON (left) and ISO-HAS-B (right) cells. Data are the mean ± SD of three independent experiments. ***$P < 0.001$.

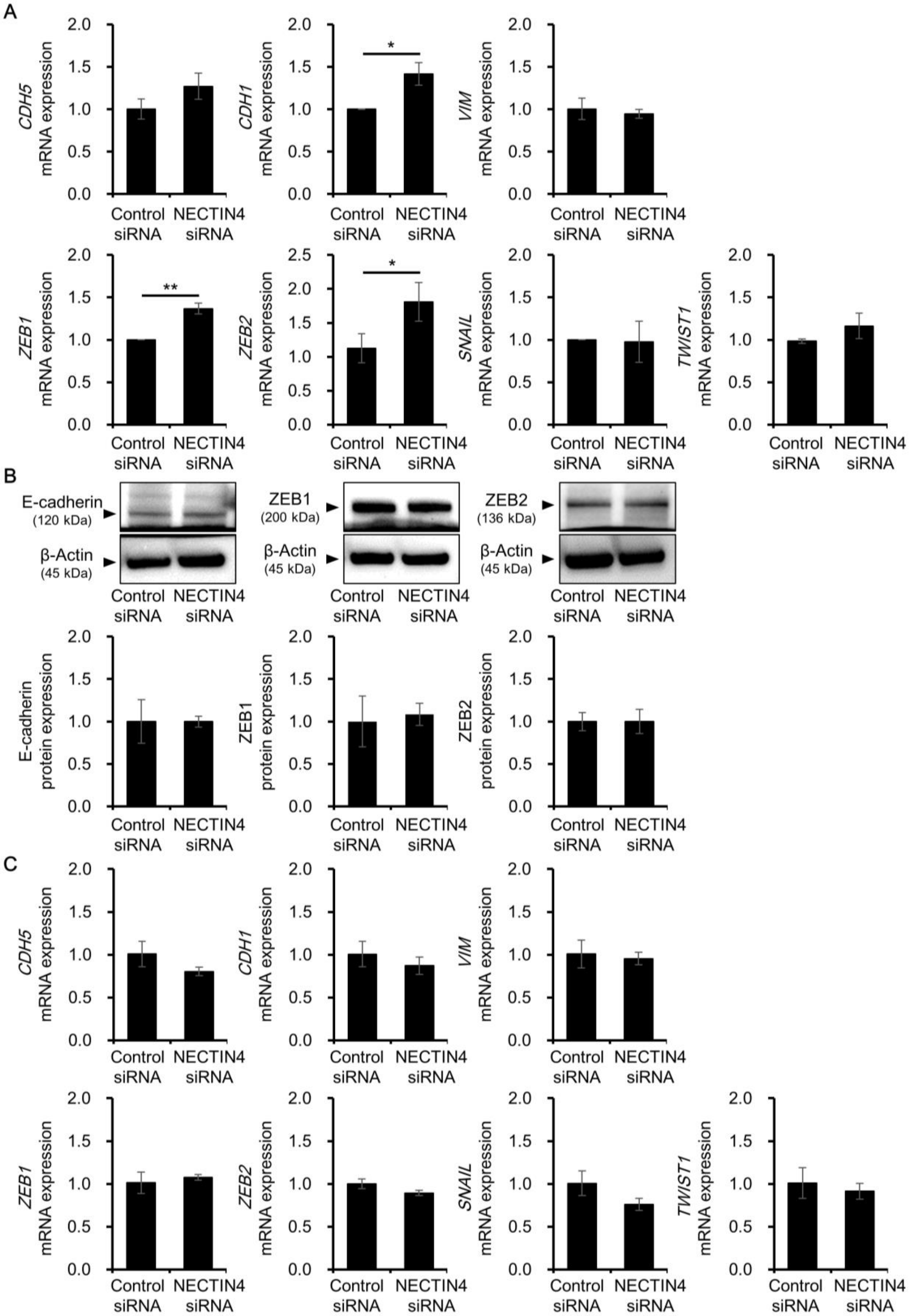

Supplementary Figure S11. Effects of NECTIN4 inhibition on EMT-related factors. HAMON and ISO-HAS-B cells were transfected with control or NECTIN4 siRNA and assessed for the expression of

EMT-related factors. (**A**) Expression of VE-cadherin (*CDH5*), E-cadherin (*CDH1*), vimentin (*VIM*), *ZEB1*, *ZEB2*, snail family transcriptional repressor 1 (*SNAI1*), and twist family BHLH transcription factor 1 (*TWIST1*) mRNA in HAMON cells. Data are the mean ± SD of three independent experiments. *$P < 0.05$, **$P < 0.01$. (**B**) Expression of E-cadherin, ZEB1, and ZEB2 proteins. Representative blot images are shown, along with the mean (± SD) expression of three independent experiments. Unedited original blot images are shown in Supplementary Fig. S12. (**C**) Expression of *CDH5*, *CDH1*, *VIM*, *ZEB1*, *ZEB2*, *SNAI1*, and *TWIST1* mRNA in ISO-HAS-B cells. Data are the mean ± SD of three independent experiments.

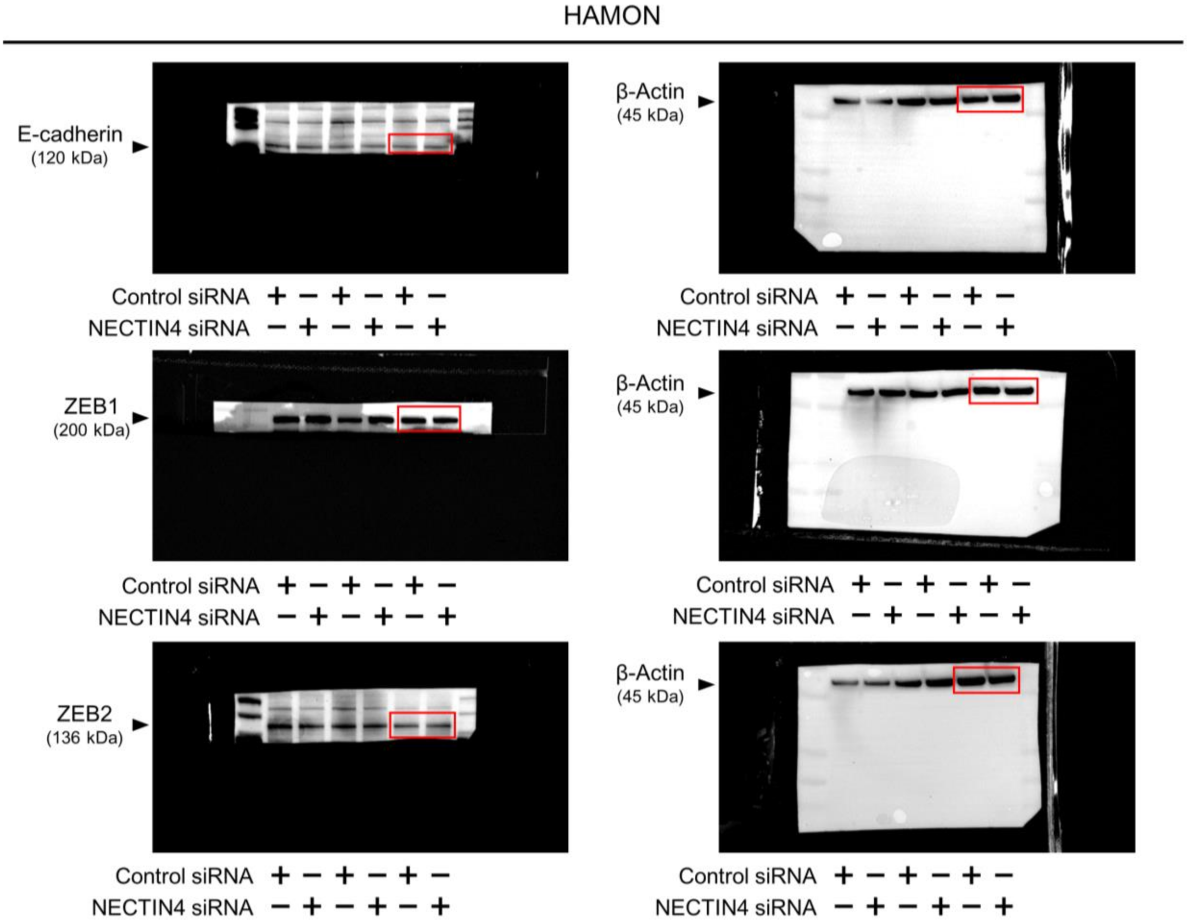

Supplementary Figure S12. Full-length blots presented in Supplementary Fig. S11B. E-Cadherin, ZEB1, and ZEB2 protein expression, along with that of β-actin, used as an internal control, in HAMON cells, as determined by western blotting. Unedited original images of blots are shown. The signal for each protein was analysed using ImageJ software and was normalized against that of β-actin. The images shown derive from the triplicate samples prepared in three independent experiments. Membranes were cut based on the size marker and hybridized with different kinds of antibodies when needed. The red boxes indicate the croped areas shown in Supplementary Fig. S11B.

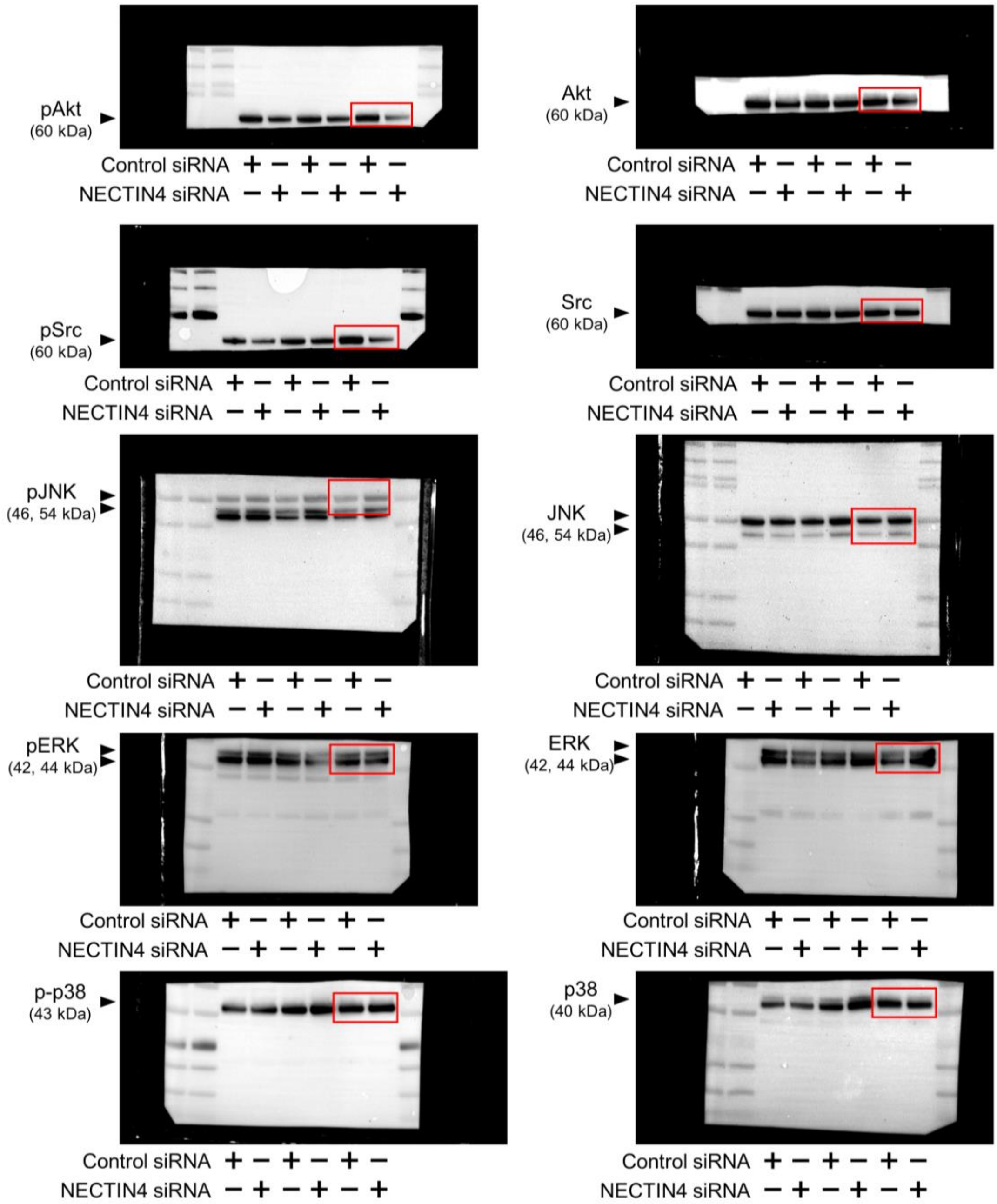

Supplementary Figure S13. Full-length blots presented in Fig. 4A for pAkt and Akt, pSrc and Src, pJNK and JNK, pERK and ERK, and p-p38 and p38. Protein expression of signaling molecules in control or NECTIN4 siRNA-transfected HAMON cells was determined by western blotting. Unedited original images of blots are shown. The signal for each phosphorylated protein was analysed using ImageJ software and divided by the signal for the total protein level of each signaling molecule. The images shown derive from the triplicate samples prepared in three independent experiments. Membranes were cut based on the size marker and hybridized with different kinds of antibodies when needed. The red boxes indicate the croped areas shown in Fig. 4A.

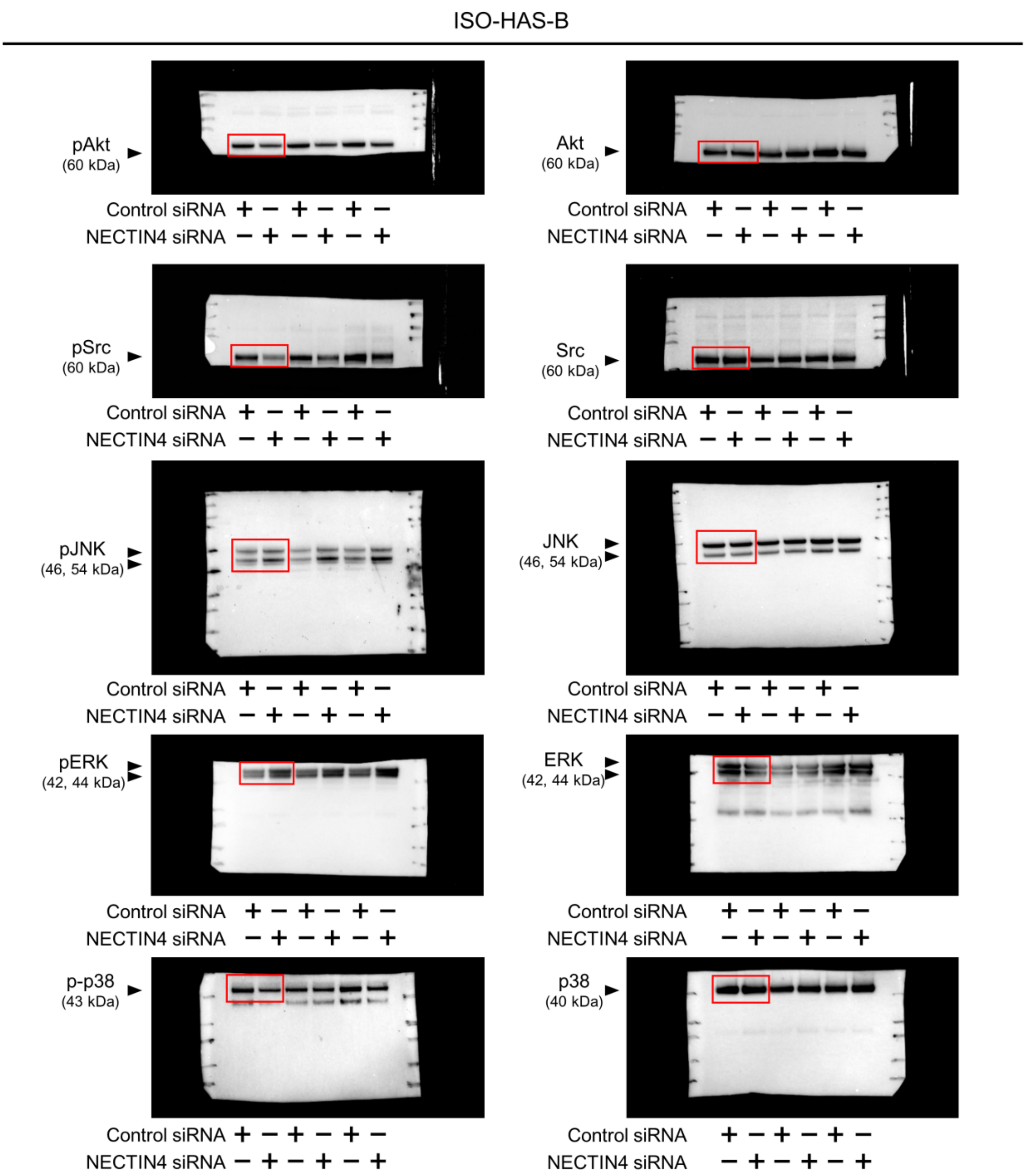

Supplementary Figure S14. Full-length blots presented in Fig. 4B for pAkt and Akt, pSrc and Src, pJNK and JNK, pERK and ERK, and p-p38 and p38. Protein expression of signaling molecules in control or NECTIN4 siRNA-transfected ISO-HAS-B cells was determined by western blotting. Unedited original images of blots are shown. The signal for each phosphorylated protein was analysed using ImageJ software and divided by the signal for the total protein level of each signaling molecule. The images shown derive from the triplicate samples prepared in three independent experiments. Membranes were cut based on the size marker and hybridized with different kinds of antibodies when needed. The red boxes indicate the croped areas shown in Fig. 4B.

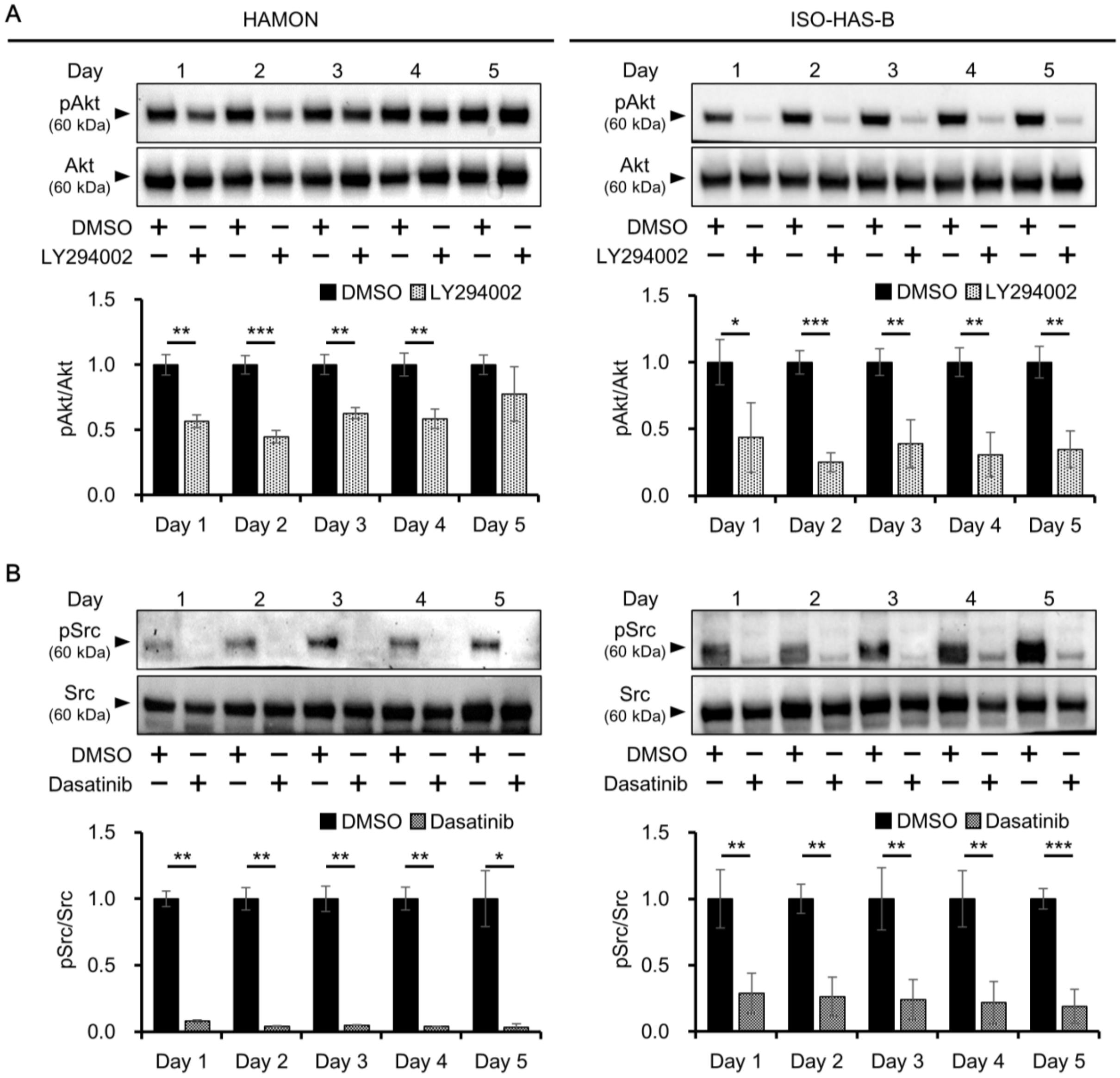

Supplementary Figure S15. Inhibition of Akt and Src phosphorylation by specific inhibitors. HAMON and ISO-HAS-B cells were treated with DMSO (0.1%), LY294002 (10 μM), or dasatinib (100 nM) for 1–5 days and the inhibition of (**A**) Akt and (**B**) Src phosphorylation was assessed. Representative blot images are shown, in addition to the mean (± SD) ratio of total to phosphorylated Akt or Src from three independent experiments. *P < 0.05, **P < 0.01, and ***P < 0.001. Unedited original blot images are shown in Supplementary Fig. S16.

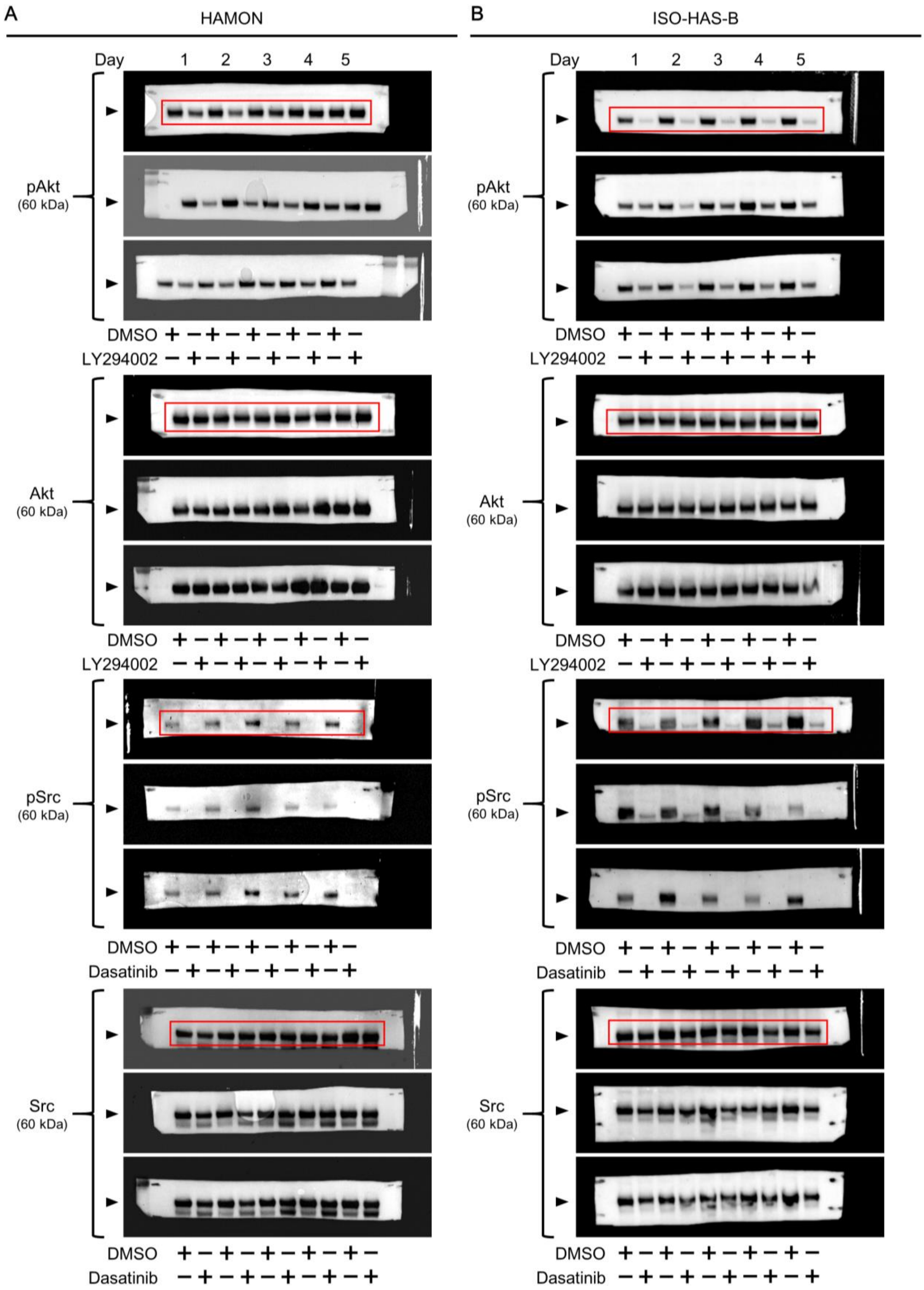

Supplementary Figure S16. Full-length blots presented in Supplementary Fig. S15. Unedited original images blots of pAkt, Akt, pSrc, and Src in (**A**) HAMON and (**B**) ISO-HAS-B cells presented in Supplementary Fig. S15. The signal for each phosphorylated protein was analysed using ImageJ software and

divided by the signal for the total protein level of each signaling molecule. The images shown derive from the three independent experiments. Membranes were cut based on the size marker and hybridized with different kinds of antibodies when needed. The red boxes indicate the croped areas shown in Supplementary Fig. S15.

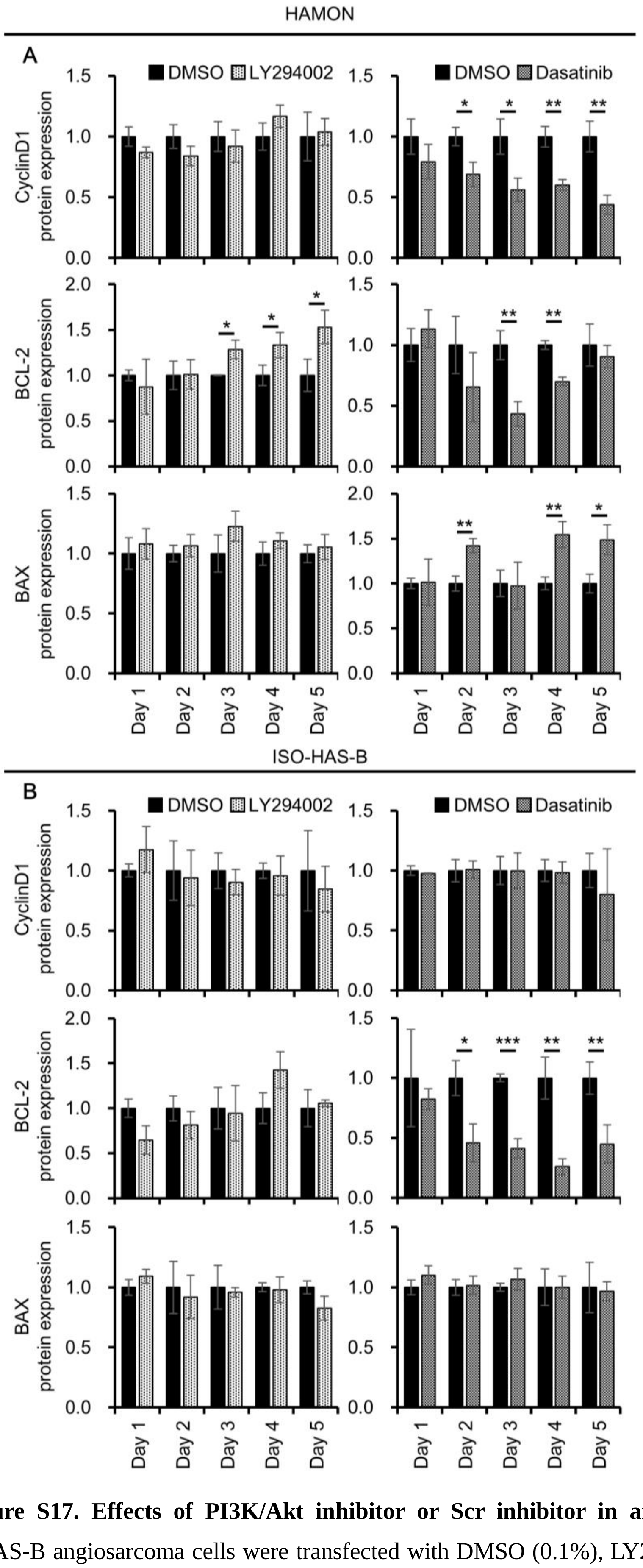

Supplementary Figure S17. Effects of PI3K/Akt inhibitor or Scr inhibitor in angiosarcoma cells. HAMON and ISO-HAS-B angiosarcoma cells were transfected with DMSO (0.1%), LY294002 (10 μM), or

dasatinib (100 nM) for 1–5 days. (**A,B**) Protein expressions of cyclin D1, BCL-2, and BAX. Mean (± SD) cyclin D1, BCL-2, and BAX expressions at the protein levels in (**A**) HAMON and in (**B**) ISO-HAS-B determined in three independent experiments. *$P < 0.05$, **$P < 0.01$, and ***$P < 0.001$.

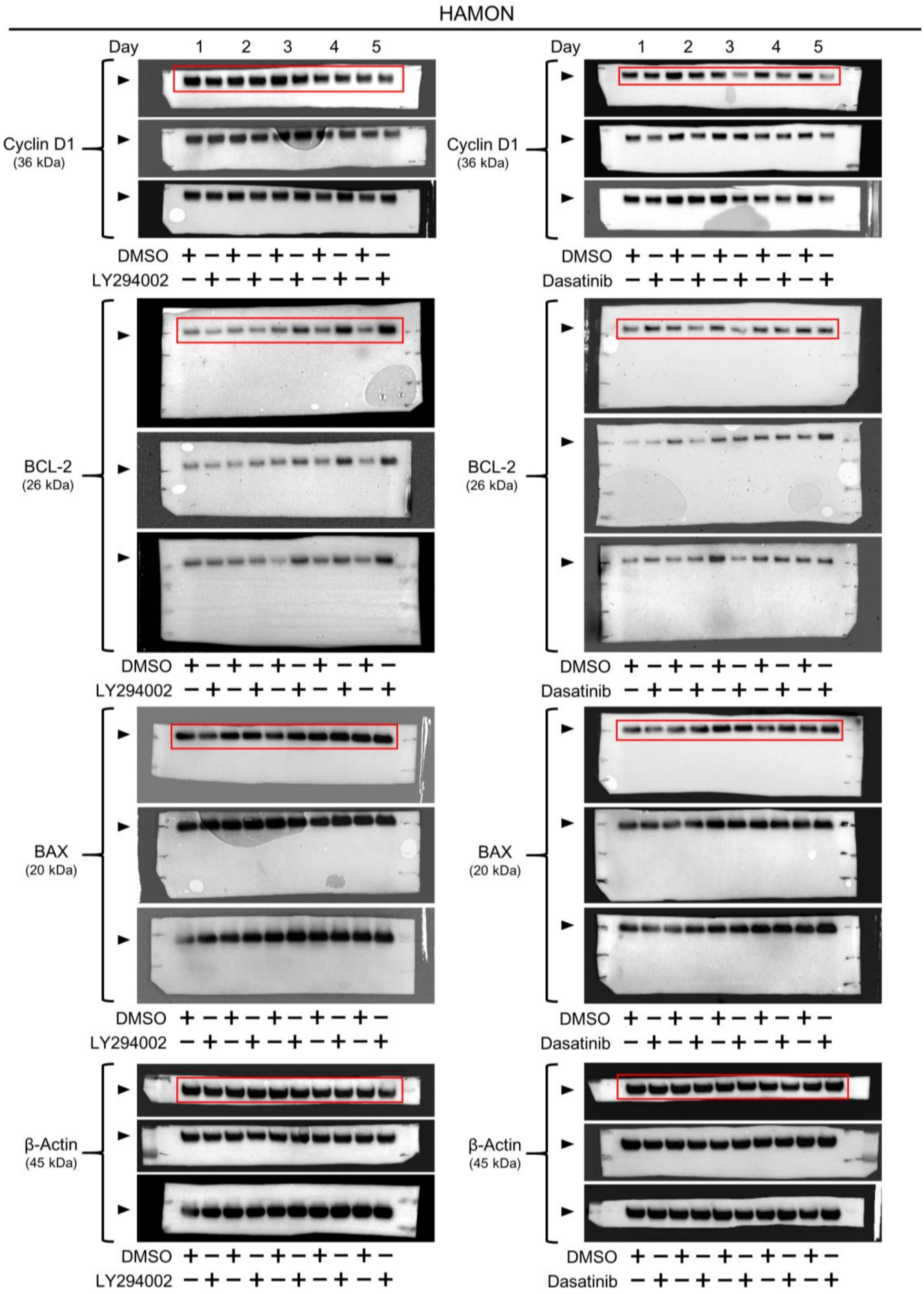

Supplementary Figure S18. Full-length blots presented in Fig. 4E. Cyclin D1, BCL-2, BAX, and β-actin protein expression in HAMON cells was determined by western blotting. Unedited original images are of those shown in Fig. 4E, upper (LY294002-treated) and Fig. 4E, lower (dasatinib-treated). The signal for each protein was analysed using ImageJ software and was normalized against that of β-actin. The images shown derive from the triplicate experiments. Membranes were cut based on the size marker and hybridized with different kinds of antibodies when needed. The red boxes indicate the croped areas shown in Fig. 4E.

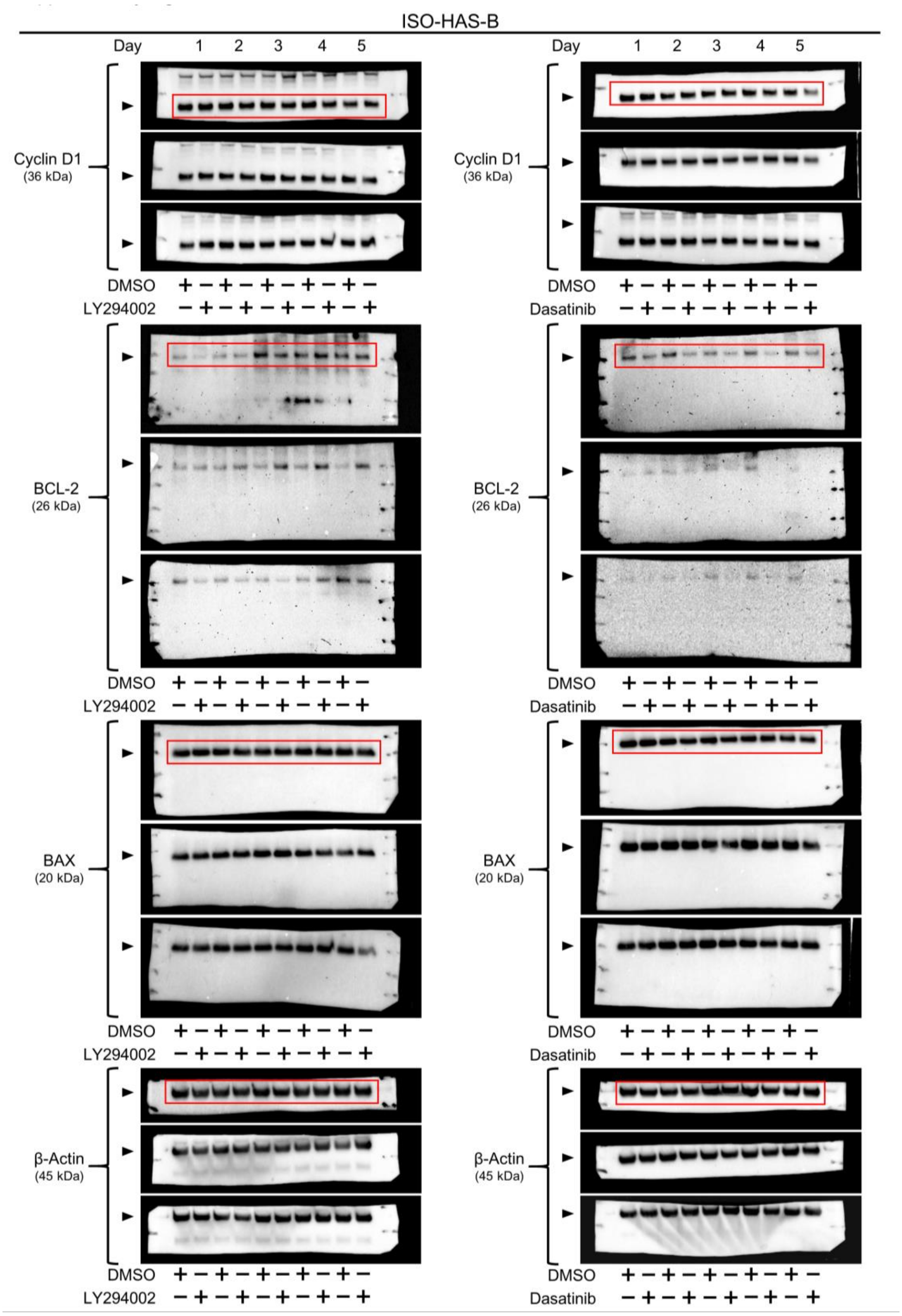

Supplementary Figure S19. Full-length blots presented in Fig. 4F. Cyclin D1, BCL-2, BAX, and β-actin protein expression in ISO-HAS-B cells was determined by western blotting. Unedited original images are of those shown in Fig. 4F, upper (LY294002-treated) and Fig. 4F, lower (dasatinib-treated). The signal for each protein was analysed using ImageJ software and was normalized against that of β-actin. The images shown derive from the triplicate experiments. Membranes were cut based on the size marker and hybridized with different kinds of antibodies when needed. The red boxes indicate the croped areas shown in Fig. 4F.

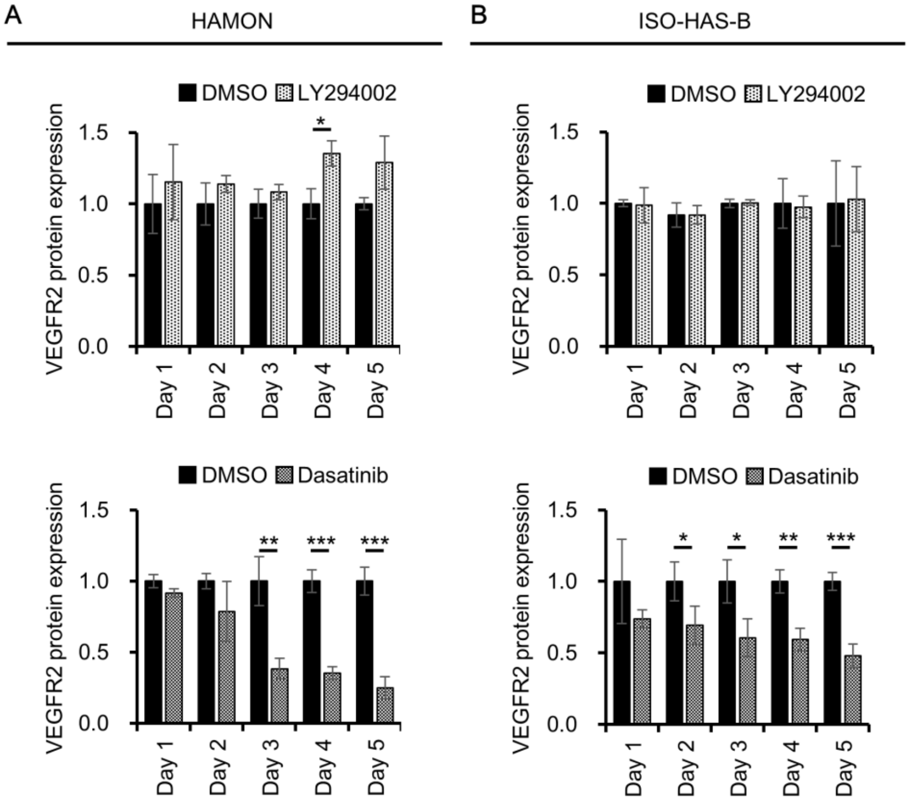

Supplementary Figure S20. Effect of PI3K/Akt inhibitor or Scr inhibitor in angiosarcoma cells. HAMON and ISO-HAS-B angiosarcoma cells were treated with DMSO (0.1%), LY294002 (10 μM), or dasatinib (100 nM) for 1–5 days. (**A,B**) Protein expressions ofVEGFR2. Mean (± SD) VEGFR2 expression at the protein levels in (**A**) HAMON and in (**B**) ISO-HAS-B determined in three independent experiments. **P* < 0.05, ***P* < 0.01, and ****P* < 0.001.

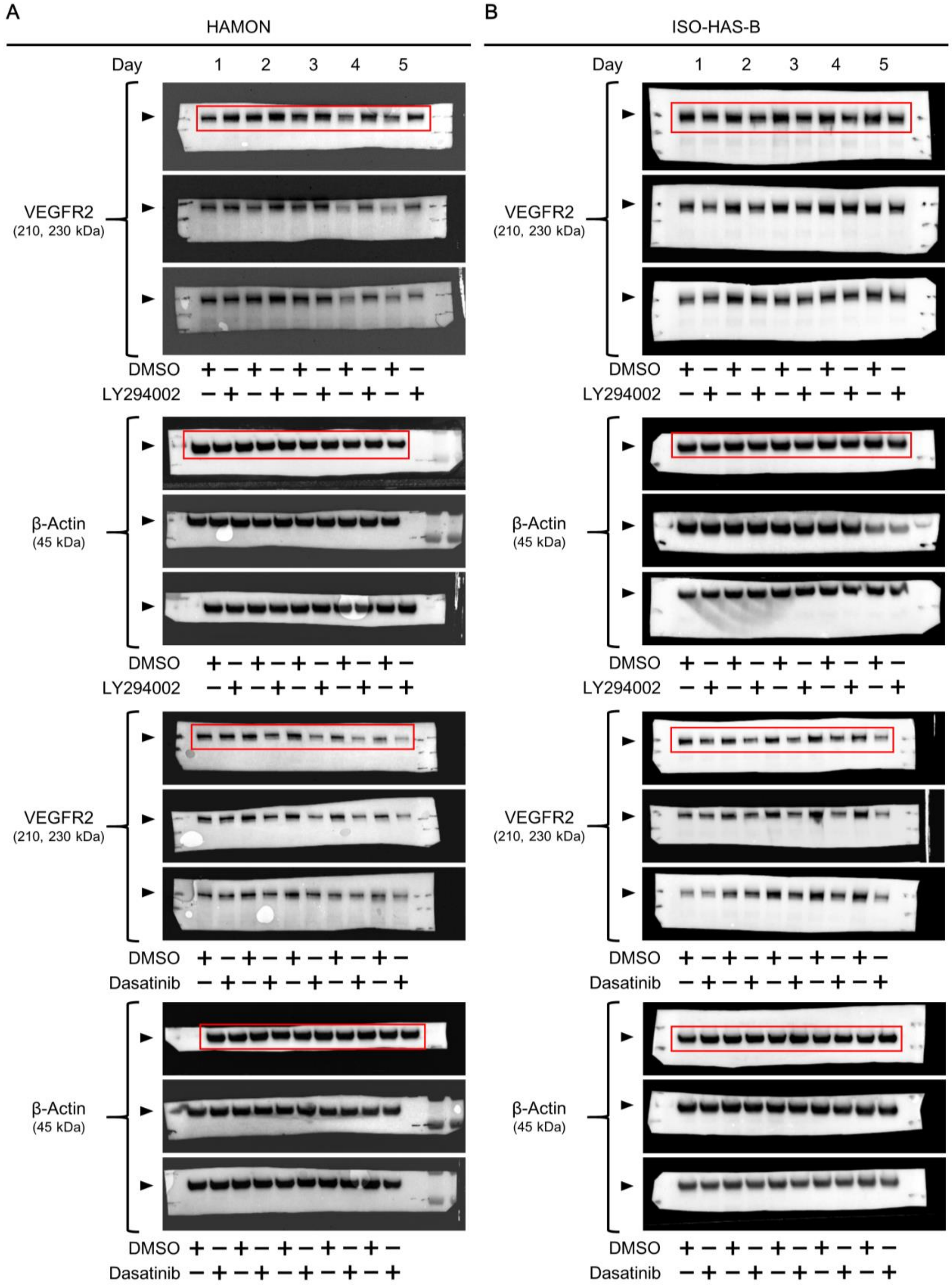

Supplementary Figure S21. Full-length blots presented in Fig. 5C and 5D. VEGFR2 and β-actin protein expression in HAMON and ISO-HAS-B cells was determined by western blotting. (**A**) Unedited original images are of those shown in Fig. 5C, upper (LY294002-treated) and Fig. 5C, lower (dasatinib-treated) in HAMON cells. (**B**) Unedited original images are of those shown in Fig. 5D, upper (LY294002-treated) and

Fig. 5D, lower (dasatinib-treated) in ISO-HAS-B cells. The signal for each protein was analysed using ImageJ software and was normalized against that of β-actin. The images shown derive from the triplicate experiments. Membranes were cut based on the size marker and hybridized with different kinds of antibodies when needed. The red boxes indicate the croped areas shown in Fig. 5.

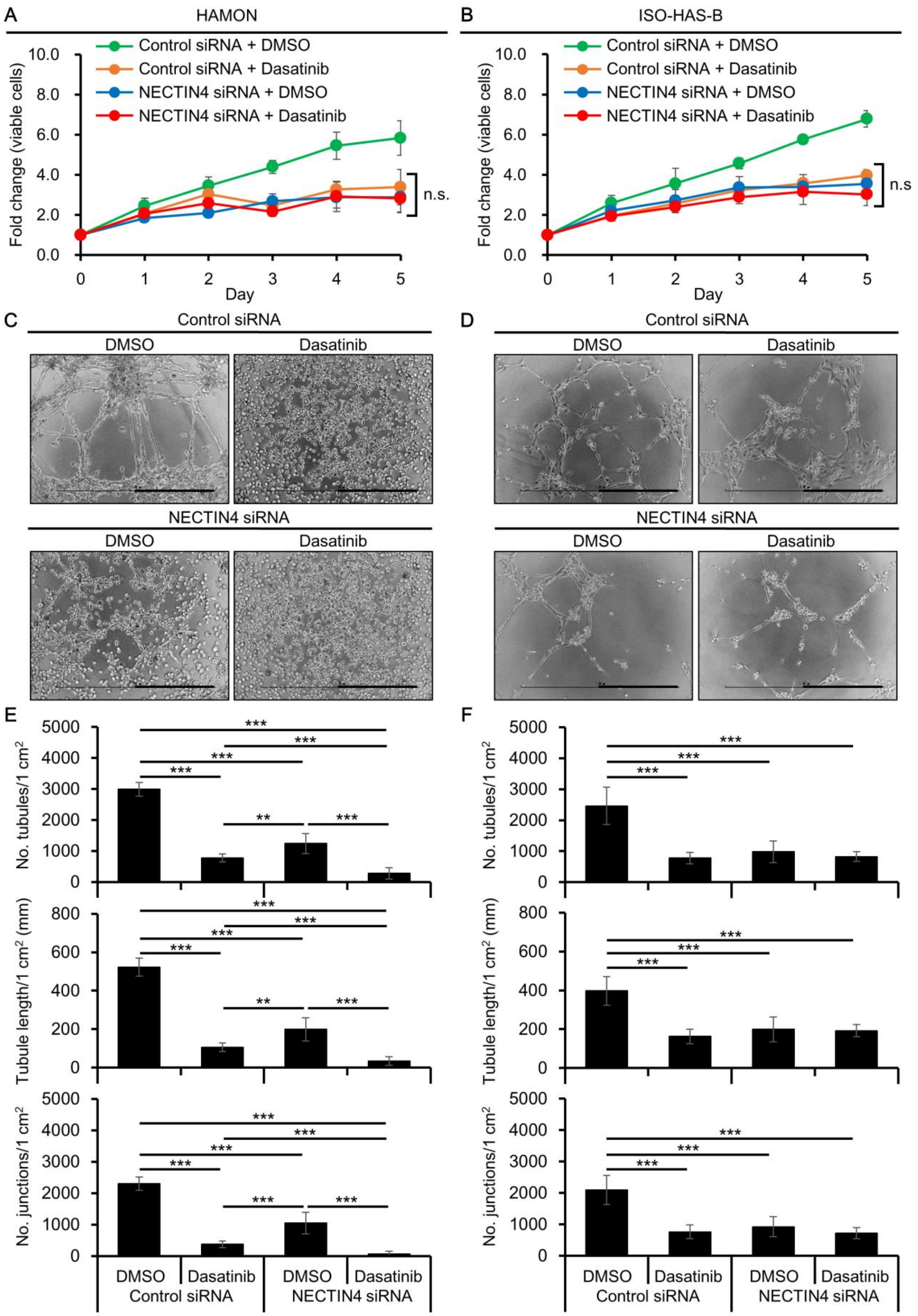

Supplementary Figure S22. Effects of dasatinib in NECTIN4 siRNA-transfected angiosarcoma cells. Control or NECTIN4 siRNA-transfected HAMON and ISO-HAS-B cells were further treated with dasatinib

and assessed for cell proliferation and angiogenesis. (**A,B**) Mean (± SD) number of viable cells in siRNA-transfected, DMSO (0.1%) or dasatinib (100 nM)-treated (**A**) HAMON and (**B**) ISO-HAS-B cells, as detected by the CCK-8 assay. Data show fold changes relative to Day 0. Experiments were repeated three times, with three wells used for each condition. n.s.; no significant differences between NECTIN4-siRNA transfected DMSO-treated cells and NECTIN4-siRNA transfected dasatinib-treated cells. (**C,D**) Representative images of angiogenesis in (**C**) HAMON and (**D**) ISO-HAS-B cells. Scale bars = 0.5 mm. (**E,F**) Number of tubules per 1 cm^2, tubule length per 1 cm^2, and the number of junctions per 1 cm^2 of (**E**) HAMON and (**F**) ISO-HAS-B cells in the angiogenesis assay. Data are the mean ± SD of three independent experiments. ***P* < 0.01 and ****P* < 0.001.

Supplementary Table S1. Clinicopathological factors associated with NECTIN4 expression

Parameters	NECTIN4 expression		p-Value
	Negative	Positive	
**Age (years)**			
	71.15 ± 12.72	69.15 ± 11.52	0.4917
**Sex**			
Male	30	13	0.2257
Female	17	14	
**Primary tumor site**			
Skin	36	15	0.0723
Non-skin	11	12	
**Tumor subtype**			
Non-epithelioid	42	19	0.0568
Epithelioid	5	8	

Supplementary Table S2. Primer sequences

Gene symbol	Primer sequence
ACTB	Sense: 5′-ATTGCCGACAGGATGCAGA-3′
	Antisense: 5′-GAGTACTTGCGCTCAGGAGGA-3′
BAX	Sense: 5′-GGACGAACTGGACAGTAACATGG-3′
	Antisense: 5′-GCAAAGTAGAAAAGGGCGACAAC-3′
BCL2	Sense: 5′-ATCGCCCTGTGGATGACTGAG-3′
	Antisense: 5′-CAGCCAGGAGAAATCAAACAGAGG-3′
CCND1	Sense: 5′-GCTGCGAAGTGGAAACCATC-3′
	Antisense: 5′-CCTCCTTCTGCACACATTTGAA-3′
CDH1	Sense: 5′-TGCCCAGAAAATGAAAAAGG-3′
	Antisense: 5′-GTGTATGTGGCAATGCGTTC-3′
CDH5	Sense: 5′-GAAGCCTCTGATTGGCACAGTG-3′
	Antisense: 5′-TTTTGTGACTCGGAAGAACTGGC-3′
NECTIN4	Sense: 5′-CAAAATCTGTGGCACATTGG-3′
	Antisense: 5′-GCTGACATGGCAGACGTAGA-3′
SNAIL	Sense: 5′-GCCTAGCGAGTGGTTCTTCT-3′
	Antisense: 5′-TAGGGCTGCTGGAAGGTAAA-3′
TWIST1	Sense: 5′-AAGGCATCACTATGGACTTTCTCT-3′
	Antisense: 5′-GCCAGTTTGATCCCAGTATTTT-3′
VEGFA	Sense: 5′-GCCTTGCCTTGCTGCTCTAC-3′
	Antisense: 5′-TGATTCTGCCCTCCTCCTTCTG-3′
$VEGF_{165}$	Sense: 5′-GAGCGGAGAAAGCATTTGTT-3′
	Antisense: 5′-CTCGGCTTGTCACATCTGC-3′
VEGFR2	Sense: 5′-GGACTCTCTCTGCCTACCTCAC-3′
	Antisense: 5′-GGCTCTTTCGCTTACTGTTCTG-3′
VIM	Sense: 5′-GAGAACTTTGCCGTTGAAGC-3′
	Antisense: 5′-GCTTCCTGTAGGTGGCAATC-3′
ZEB1	Sense: 5′-GCACCTGAAGAGGACCAGAG-3′
	Antisense: 5′-TGCATCTGGTGTTCCATTTT-3′
ZEB2	Sense: 5′-TTTCAGGGAGAATTGCTTGA-3′
	Antisense: 5′-CACATGCATACATGCCACTC-3′

Supplementary Table S3. Antibodies used for western blotting

Antibody	Host	Manufacturer	Catalog no. (RRID)	Dilution
Anti-human β-actin	Rabbit	Cell Signaling Technologies	#4970 (AB_223172)	1:2,000
Anti-BAX antibody	Rabbit	Cell Signaling Technologies	#2772 (AB_10695870)	1:1000
Anti-Bcl-2 antibody	Rabbit	Cell Signaling Technologies	#4223 (AB_1903909)	1:500
Anti-human cyclin D1	Rabbit	Cell Signaling Technologies	#55506 (AB_28227374)	1:500
Anti-E-cadherin antibody	Mouse	BD Biosciences	610181 (AB_397580)	1:5,000
Anti-human NECTIN4	Rabbit	Abcam	ab192033	1:1,000
Anti-human VEGFR2	Rabbit	Cell Signaling Technologies	#2479 (AB_2212507)	1:1000
Anti-human ZEB1	Rabbit	Abcam	ab155249	1:1,000
Anti-human ZEB2	Rabbit	Abcam	ab138222 (AB_2801551)	1:1,000
Anti-Akt	Rabbit	Cell Signaling Technologies	#9272 (AB_329827)	1:1,000
Anti-pAkt	Rabbit	Cell Signaling Technologies	#4969 (AB_2315049)	1:2,000
Anti-ERK	Rabbit	Cell Signaling Technologies	#9102 (AB_330744)	1:1,000
Anti-pERK	Rabbit	Cell Signaling Technologies	#4370 (AB_2315112)	1:2,000
Anti-JNK	Rabbit	Cell Signaling Technologies	#9258 (AB_2141027)	1:1,000
Anti-pJNK	Rabbit	Cell Signaling Technologies	#4668 (AB_823588)	1:1,000
Anti-p38 MAPK	Rabbit	Cell Signaling Technologies	#8690 (AB_10999090)	1:1,000
Anti-p-p38 MAPK	Rabbit	Cell Signaling Technologies	#4511 (AB_2139682)	1:1,000
Anti-Src	Rabbit	Cell Signaling Technologies	#2123 (AB_2106047)	1:1,000
Anti-pSrc	Rabbit	Cell Signaling Technologies	#2101 (AB_331697)	1:1,000
Anti-mouse IgG HRP	Horse	Cell Signaling Technologies	#7076 (AB_330924)	1:10,000
Anti-rabbit IgG HRP	Goat	Cell Signaling Technologies	#7074 (AB_2099233)	1:10,000

HRP, horseradish peroxidase.
